# Deferoxamine Inhibits Canine Parvovirus by Suppressing Ferroptosis and Viral Replication

**DOI:** 10.3390/vetsci12121192

**Published:** 2025-12-12

**Authors:** Kai Yu, Haoyuan Ma, Siqi Zhang, Jiawei Zhao, Jingrui Hao, Jialiang Xie, Hao Yu, Jianyou Jin, Xinpeng Ji, Shuoning Cao, Zheng Sun, Shujiang Xue, Shengwei Ji, Zhiqiang Xu, Chenghui Li, Rui Du, Xu Gao

**Affiliations:** Department of Veterinary Medicine, College of Agricultural, Yanbian University, Yanji 133002, China; 13044365811@163.com (K.Y.); bakougenn@gmail.com (H.M.); zsq18953192389@outlook.com (S.Z.); 17519267465@163.com (J.Z.); m17808001838@163.com (J.H.); 13040368086@163.com (J.X.); 13009151929@163.com (H.Y.); 13943371101@163.com (J.J.); j1596078301@163.com (X.J.); csn17767895339@outlook.com (S.C.); 17649816000@163.com (Z.S.); sjxue@ybu.edu.cn (S.X.); jishengwei0903@hotmail.com (S.J.); 0000008040@ybu.edu.cn (Z.X.); lichenghuivip0056@163.com (C.L.)

**Keywords:** canine parvovirus, ferroptosis, deferoxamine, transferrin receptor

## Abstract

CPV is a highly pathogenic virus that enters host cells via the TFR, but its pathogenic mechanisms are not fully understood. In this study, we demonstrated that CPV infection induces ferroptosis in CRFK cells, characterized by elevated lipid peroxidation, increased intracellular ferrous iron, a reduced GSH/GSSG ratio, and mitochondrial damage. At the molecular level, CPV downregulated the ferroptosis-inhibiting proteins GPX4 and FTH1 while upregulating NCOA4 and Drp1. Notably, treatment with the iron chelator DFO both inhibited viral replication and attenuated CPV-induced ferroptosis. Collectively, our findings identify ferroptosis as a critical pathogenic mechanism of CPV infection and highlight the therapeutic potential of DFO in controlling CPV by restoring iron homeostasis. These insights provide a mechanistic basis for the development of ferroptosis-targeted antiviral strategies against CPV.

## Simple Summary

We found that canine parvovirus (CPV) infection triggered a cell death called ferroptosis. Viruses damage cells and promote their own replication by disrupting the iron balance in cells, leading to the accumulation of toxic substances. Importantly, we found that a drug called deferoxamine (DFO) can inhibit ferroptosis by removing excess iron and effectively block viral replication. This provides a novel potential strategy for the treatment of CPV infection.

## 1. Introduction

Canine parvovirus disease (CP) is a highly contagious acute hemorrhagic disease caused by CPV. The main clinical manifestation of this disease is viral enteritis type, characterized by severe vomiting, bloody diarrhea, and a significant reduction in white blood cells. In puppies, CPV often leads to myocarditis or mixed enteritis and myocarditis infections, with rapid disease progression. If not intervened in time, the mortality rate in the later stage of infection can be over 90%, posing a serious threat to dogs, especially puppies [1,2,3,4]. CPV belongs to the Parvoviridae family and the parvovirus genus, which is a non-enveloped single-stranded DNA virus [5,6]. Its genome is about 5 kb in length and contains two major open reading frames (ORFs), encoding nonstructural and structural proteins, respectively [7]. Among the structural proteins of the virus, the VP2 protein is the most important and abundant component, accounting for about 90% of the total viral capsid protein. VP2 protein specifically binds to TFR on the surface of host cells during virus infection, mediating virus entry into cells and initiating virus replication [8,9]. Previous studies have shown that CPV infection can induce apoptosis in host cells in vitro [10,11,12]. However, whether CPV can also trigger ferroptosis has not yet been investigated.

In recent years, ferroptosis has emerged as a novel form of regulated cell death that has attracted considerable attention in the study of diverse pathophysiological processes [13,14]. It is characterized by iron-dependent lipid peroxidation and the excessive accumulation of reactive oxygen species (ROS), mechanistically and morphologically distinct from apoptosis, necrosis, and autophagy [15,16]. At the molecular level, disruption of intracellular iron homeostasis leads to iron overload, which catalyzes the Fenton reaction to generate highly reactive hydroxyl radicals (OH•) [17]. These radicals cause irreversible damage, including lipid peroxidation, mitochondrial shrinkage, and plasma membrane rupture, ultimately triggering ferroptosis [18,19]. An increasing number of studies have shown that many viruses can induce ferroptosis in host cells through multiple mechanisms to promote their own replication [20], HCV can induce GPX4 expression through NS5A, and HCMV uses USP24 in combination with pUL38 to regulate NCOA4 and reduce ferritin autophagy, These findings suggest that intervention of ferroptosis signaling pathway is an important strategy for viruses to establish infection in the host, and targeting ferroptosis may provide a new perspective for antiviral therapy [21,22].

As a transferrin receptor on the cell surface, TFR is the core molecule of iron uptake in cells. Previous studies have found that coxsackievirus B3 (CVB3), influenza A virus (IAV), and murine mammary tumor virus (MMTV) can also use TFR to enter host cells and disrupt intracellular iron homeostasis and eventually lead to ferroptosis. This provides a unique reason to investigate the possibility that CPV infection may interfere with iron homeostasis and induce ferroptosis [23,24,25].

Numerous experimental model studies have confirmed that DFO is an efficient and highly specific iron chelating agent [26,27]. DFO has a high affinity for free Fe^2+^/Fe^3+^, which can significantly reduce the level of intracellular labile iron pool (LIP) by 40–60%, thereby effectively blocking the Fenton reaction and upregulating ferritin expression by approximately 2–3-fold, promoting the safe storage of iron [28,29]. Notably, the antiviral potential of DFO has been demonstrated in a variety of viruses. For example, DFO inhibits the replication of human cytomegalovirus (HCMV) in infected human foreskin fibroblast (HFF) cultures at concentrations without significant adverse effects in humans. In addition, DFO also showed inhibitory activity against human immunodeficiency virus (HIV) [30,31]. Given that CPV is dependent on TFR to mediate invasion, it is possible that CPV directly interferes with the normal physiological function of TFR, thereby initiating an imbalance in intracellular iron homeostasis. This imbalance, manifested as iron accumulation and impaired antioxidant defense systems, may in turn activate key executive pathways of ferroptosis. We hypothesized that DFO may exert its effect of inhibiting viral replication while regulating iron metabolism by chelating intracellular iron ions.

Therefore, the aim of this study was to investigate whether CPV infection induces ferroptosis by disturbing intracellular iron homeostasis, and to further evaluate whether iron chelator DFO can be used as a candidate therapeutic drug to inhibit viral replication and restore iron homeostasis [26]. The results of this study not only provide a new perspective on the pathogenic mechanism of CPV but also suggest that targeting the ferroptosis pathway may be a promising antiviral strategy in the veterinary field.

## 2. Materials and Methods

### 2.1. Cell Line and Virus

CRFK (Crandell-Rees feline kidney) cells were obtained from Zhejiang Baidi Biotechnology Co., Ltd., Hangzhou, China and cultured at 37 °C in a humidified incubator with 5% CO_2_. Cells were maintained in Dulbecco’s Modified Eagle Medium (DMEM; Baidi, Hangzhou, China) supplemented with 10% fetal bovine serum (FBS; Baidi, Hangzhou, China) and 1% penicillin–streptomycin (Solarbio, Beijing, China). The CPV-YBYJ strain (2 × 10^7^ TCID_50_/mL) preserved in our laboratory (GenBank accession no. NC_001806) was propagated in CRFK cells and used for subsequent experiments at a multiplicity of infection (MOI) of 1 unless otherwise indicated.

CRFK cells were used because they are a permissive and widely adopted in vitro model for CPV infection. CPV utilizes TFR for entry, and feline TFR expressed on CRFK cells supports productive CPV replication, enabling mechanistic studies. Moreover, the ferroptosis-related pathways examined here are conserved across mammalian species.

### 2.2. RNA Interference

Small interfering RNA (siRNA) targeting the feline TFR1 gene was designed based on the Felis catus TFR coding sequence (GenBank accession no.: NC_058376.1). The siRNA (siTFR) sequence was as follows: 5′-CCACUGUUGUAUUCGCUUAUU-3′. A non-targeting siRNA (siNC) with no homology to any feline gene was used as a negative control (Comate Bioscience Co., Ltd., Jilin, China). CRFK cells were transfected with 50 nM siTFR or negative control siRNA (siNC) using Lipofectamine™ 2000 (Invitrogen, Carlsbad, CA, USA) according to the manufacturer’s protocol. At 24 h post-transfection, CPV was used to infect cells with a complex infection number (MOI) of 1. Samples were collected 48 h after transfection for subsequent testing.

### 2.3. qRT-PCR

Total RNA was extracted from CPV-infected CRFK cells using TRIzol reagent (Invitrogen, Carlsbad, CA, USA). One microgram of RNA was reverse transcribed into complementary DNA (cDNA) using SuperScript III Reverse Transcriptase (Invitrogen, USA) according to the manufacturer’s instructions. Quantitative PCR (qPCR) was performed in a final volume of 20 μL containing 1 × SYBR Green PCR Master Mix (Thermo Fisher Scientific, Waltham, MA, USA), 0.25 μM forward primer and 0.25 μM reverse primer, and 1 μL cDNA template (equivalent to cDNA from 50 ng RNA), with nuclease-free water added to the volume. The amplification program consisted of an initial denaturation at 95 °C for 2 min, followed by 40 cycles of 95 °C for 10 s and 60 °C for 15 s. Primer sequences are listed in Table S1, and relative gene expression levels were calculated using the 2^−ΔΔCT^ method.

### 2.4. Transmission Electron Microscopy (TEM) Examination

CRFK cells were seeded in six-well plates and infected with CPV at a multiplicity of infection (MOI) of 1 when they reached 80% confluence. Once evident cytopathic effects (CPE) were observed, the cells were harvested by trypsinization and centrifuged at 1000× *g* for 5 min. Cell pellets were fixed in 2.5% glutaraldehyde and post-fixed with 1% osmium tetroxide. The samples were dehydrated through a graded acetone series, infiltrated with a 1:1 mixture of ethanol and epoxy resin at room temperature for 7 h, embedded in 100% Epon resin, and polymerized at 60 °C for 24 h. Ultrathin sections (80 ± 5 nm) were cut using an ultramicrotome, stained with 4% uranyl acetate and lead citrate, and examined under a transmission electron microscope (TEM).

### 2.5. Lipid Peroxidation Assay (MDA Measurement)

Intracellular malondialdehyde (MDA) levels, as an indicator of lipid peroxidation, were measured using a commercial MDA assay kit (Beyotime Biotechnology, Shanghai, China) according to the manufacturer’s instructions. Briefly, cell lysate supernatants were collected and reacted with thiobarbituric acid (TBA), forming an MDA-TBA adduct that generates a chromogenic product measurable at 535 nm. The absorbance at 535 nm, which is directly proportional to the MDA concentration, was measured using a microplate reader (DetieBio, Nanchang, China). The results were expressed as nmol MDA per mg of protein. Each assay included a reagent blank (no sample) and serially diluted standards as internal controls. Detailed calculation formulas are provided in the Supplementary Methods S1.

### 2.6. Assay of GSH/GSSG Levels

Intracellular reduced glutathione (GSH) and oxidized glutathione (GSSG) levels were measured using a commercial GSH/GSSG Assay Kit (Beyotime Biotechnology, Shanghai, China) according to the manufacturer’s instructions. Briefly, cell lysates were prepared and supernatants collected. Total glutathione (GSH + GSSG) and GSSG were quantified following the kit protocol, and absorbance was recorded at 412 nm using a microplate reader (DetieBio, Nanchang, China). GSH levels were calculated by subtracting GSSG from total glutathione. The result was normalized to protein content and expressed as nmol/mg protein. Blank reactions and standard controls were included for calibration. Detailed calculation formulas are provided in the Supplementary Methods S2.

### 2.7. Ferrous Iron Detection

Intracellular ferrous iron (Fe^2+^) levels were measured using a commercial Ferrous Iron Colorimetric Assay Kit (Beyotime Biotechnology, Shanghai, China) following the manufacturer’s protocol. Briefly, cell lysate supernatants were collected and mixed with 1 M hydrochloric acid, after which the samples were incubated with the chromogenic reagent provided in the kit. Absorbance was recorded at 593 nm using a microplate reader (DetieBio, Nanchang, China), and Fe^2+^ concentrations were calculated based on the standard curve. The results were normalized to the total protein content and expressed as nmol/mg protein.

### 2.8. ROS Measurement

Intracellular reactive oxygen species (ROS) levels were determined using a commercial ROS detection kit (Beyotime Biotechnology, Shanghai, China). CRFK cells were seeded in 6-well plates and infected with CPV. At the indicated time points, cells were incubated with dihydroethidium (DHE) probe diluted 1:10,000 in serum-free medium at 37 °C for 30 min in the dark. Excess dye was removed by washing with PBS. Fluorescent signals were captured using an inverted fluorescence microscope (Nikon Eclipse Ti Nikon Corporation, Tokyo, Japan), and fluorescence intensity was quantified using ImageJ software, version 1.53 (National Institutes of Health, Bethesda, MD, USA). ROS levels were expressed relative to those of the control group.

### 2.9. Cell Viability Assay

Cell viability was determined by the methyl thiazolyl tetrazolium (MTT) method (Beyotime Biotechnology, Shanghai, China). CRFK cells were seeded in 96-well plates at a cell concentration of 1 × 10^4^ cells per well. After DFO (MedChemExpress, Monmouth Junction, NJ, USA), NAC (MedChemExpress, Monmouth Junction, NJ, USA), or Erastin treatment, 20 μL of MTT reagent (5 mg/mL) was added to each well, and incubated at 37 °C for 4 h. After removing the supernatant, 100 μL of dimethyl sulfoxide (DMSO) was injected into each well. After shaking at room temperature for 10 min, the absorbance at 490 nm was measured using a microplate reader (DetieBio, Nanchang, China).

### 2.10. Western Blotting

CRFK cells were infected with CPV at different MOIs when they reached 80% confluence. At 12 h post-infection, proteins were extracted with RIPA buffer, quantified using a BCA Protein Assay Kit (Takara, Beijing, China), resolved by 12.5% SDS-PAGE, and transferred onto 0.22 µm PVDF membranes (MilliporeSigma, Burlington, MA, USA). Membranes were blocked with 5% skim milk in TBST, incubated overnight at 4 °C with primary antibodies diluted in 5% BSA/TBST, and probed with HRP-conjugated secondary antibodies for 1 h at room temperature. Primary antibodies included anti-GPX4 (CST, 59735S; 1:1000), anti-ACSL4 (Abmart, TD12141; 1:2000), anti-TFR (Affinity, AF5343; 1:2000), anti-FTH1 (Abmart, PA4412; 1:1000), anti-NCOA4 (Abmart, TD4255; 1:1000), anti-ATG5 (Affinity, DF7579; 1:2000), anti-Drp1 (Affinity, DF7037; 1:2000), and anti-β-actin (Abmart, TP70573; 1:10,000). Secondary antibodies were HRP-conjugated goat anti-mouse IgG (Abways, AB0102; 1:50,000) and HRP-conjugated goat anti-rabbit IgG (Abways, AB0106; 1:50,000). Protein bands were visualized with ECL reagents (biotides, Beijing, China) and quantified ImageJ software, version 1.53 (National Institutes of Health, Bethesda, MD, USA).

### 2.11. Statistical Analysis

All quantitative data are presented as the mean ± standard deviation (SD) from at least three independent biological replicates (*n* ≥ 3). Statistical analyses were performed using GraphPad Prism (version 9.0.0). After confirming the normal distribution of data with the Shapiro–Wilk test, differences between the two groups were assessed by an unpaired two-tailed Student’s *t*-test. For comparisons among multiple groups, one-way analysis of variance (ANOVA) was conducted. A *p*-value of less than 0.05 was considered statistically significant (* *p* < 0.05, ** *p* < 0.01, *** *p* < 0.001).

## 3. Results

### 3.1. CPV Infection Can Induce Ferroptosis in CRFK Cells

**Figure 1:**
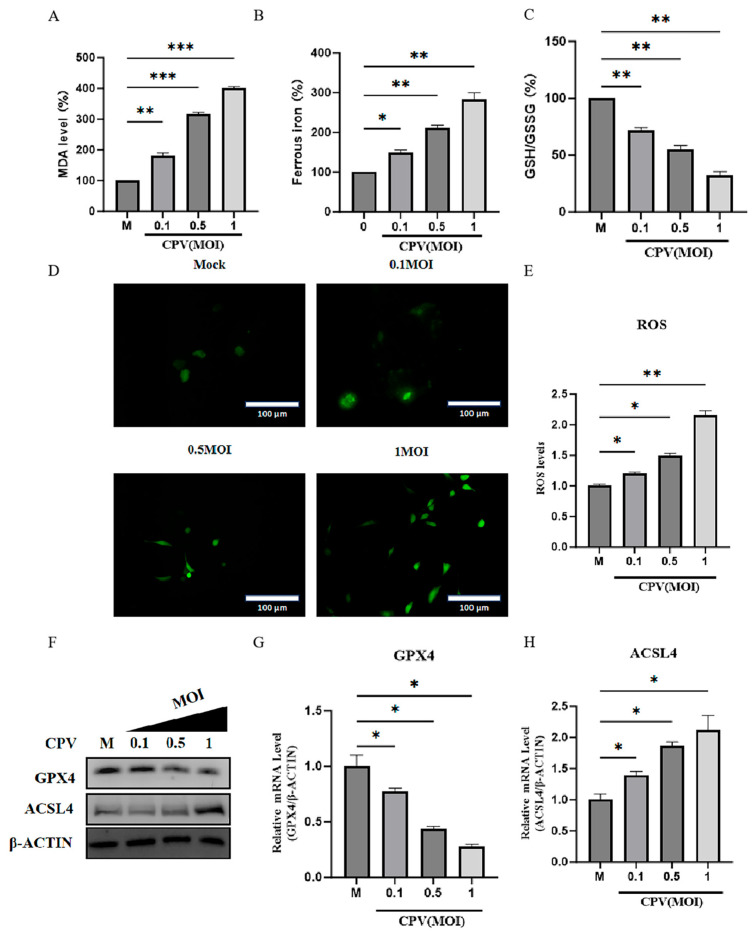
The ferroptosis of CPV infected host cells increased with the increase in virus multiplicity. (**A**) The MDA concentration in the cell lysate was detected at a wavelength of 535 nm using an MDA detection kit to assess the level of intracellular lipid peroxidation. (**B**) The concentration of ferrous iron in cell lysates was determined by an iron assay kit at a wavelength of 593 nm. (**C**) The intracellular GSH/GSSG levels were detected at a wavelength of 412 nm using the GSH/GSSG detection kit. (**D**,**E**) The intracellular ROS levels labeled with DHE probes were detected by fluorescence microscopy, green fluorescence indicates intracellular ROS detected by DHE staining. (**F**) Western Blot was used to detect the protein expression of GPX4 and ACSL4 in cells under different MOIs. (**G**,**H**) qPCR was used to analyze the expression of GPX4 and ACSL4 mRNA in cells under different MOIs. Data are presented as mean ± SD from *n* = 3 independent experiments; * *p* < 0.05, ** *p* < 0.01, *** *p* < 0.001.

To investigate whether CPV infection triggers ferroptosis, CRFK cells were infected with CPV and assessed for ferroptosis-associated markers. As the MOI increased, the intracellular MDA level (Figure 1A) and ferrous ion content (Figure 1B) significantly increased, and the intracellular GSH/GSSG level significantly decreased (Figure 1C). Lipid peroxidation is the result of excess production of iron-dependent reactive oxygen species (ROS). Accordingly, we examined ROS levels in CPV-infected cells and found that ROS content in infected cells was significantly higher with increasing virus multiplicity of infection compared to uninfected cells (Figure 1D,E). Based on the above detection, we found that compared with the uninfected cells, the mRNA and protein expression levels of GPX4 (Figure 1F,G), an important regulator of ferroptosis, were significantly decreased, and the mRNA and protein expression levels of ACSL4 (Figure 1F,H), a lipid metabolism enzyme, were significantly increased in CPV-infected cells. These results indicate that CPV infection of CRFK cells causes ferroptosis.

### 3.2. CPV Infection Disrupts Cellular Iron Homeostasis and Activates Ferroptosis-Related Signaling Pathways

**Figure 2:**
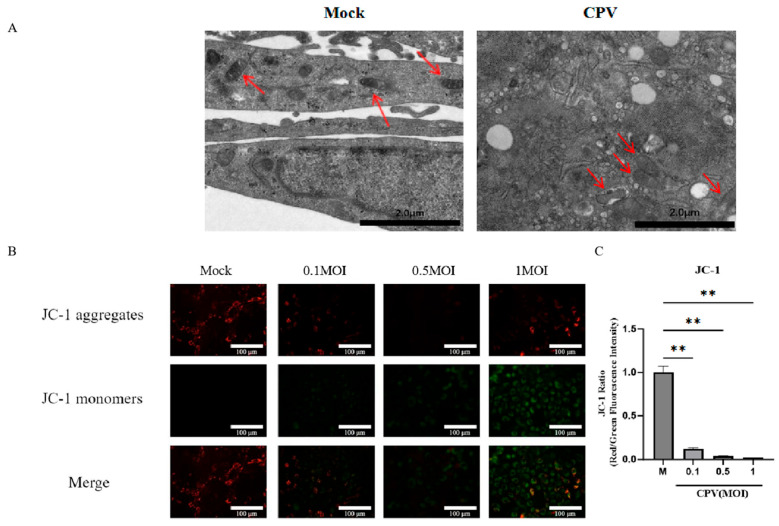
CPV infection induces ferroptosis-associated mitochondrial ultrastructural damage and loss of membrane potential in CRFK cells. (**A**) Transmission electron microscopy was used to observe the mitochondrial morphology in the control group and the CPV infection group. Representative images of normal or dysfunctional mitochondria are shown. Red arrows indicate mitochondria showing ferroptosis-associated ultrastructural changes, including swollen morphology and reduced cristae. (**B**,**C**) The level of mitochondrial membrane potential labeled with the JC-1 probe was measured by fluorescence microscopy. Red fluorescence (JC-1 aggregates) represents mitochondria with high membrane potential, whereas green fluorescence (JC-1 monomers) indicates mitochondrial depolarization. Data are presented as mean ± SD from *n* = 3 independent experiments; ** *p* < 0.01.

Another characteristic of ferroptosis is the reduction in mitochondrial cristae, rupture of the mitochondrial outer membrane, and wrinkling. Based on this, we detected the microstructure of CPV-infected cells through transmission electron microscopy. The results showed typical ultrastructural changes of ferroptosis in CRFK cells infected with CPV, including mitochondrial swelling, disrupted cristae, and increased autophagic vesicles, in contrast to the intact mitochondrial morphology observed in mock controls (Figure 2A). JC-1 staining showed a clear shift from red to green fluorescence after CPV infection, indicating mitochondrial depolarization. Quantification of the red/green fluorescence ratio confirmed a significant reduction in mitochondrial membrane potential in infected cells compared with controls (Figure 2B,C).

**Figure 3:**
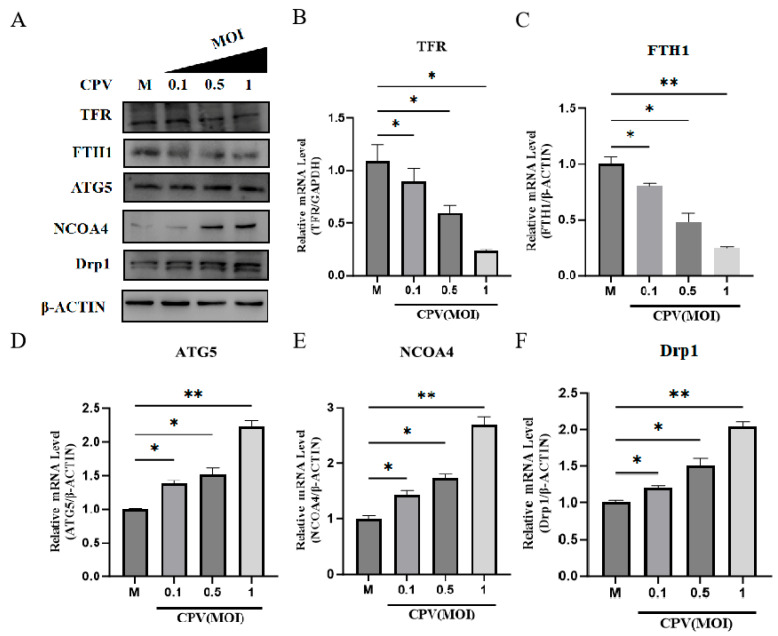
CPV infection promotes ferritin degradation. (**A**) Western Blot was used to detect the expression of TFR, FTH1, ATG5, NCOA4, and Drp1 in cells under different MOIs. (**B**–**F**) qPCR was used to analyze the expression of TFR, FTH1, ATG5, NCOA4, and Drp1 mRNA in cells under different MOIs. Data are presented as mean ± SD from *n* = 3 independent experiments; * *p* < 0.05, ** *p* < 0.01.

CPV infection significantly downregulated the expression and transcription of TFR and ferritin heavy chain 1 (FTH1) (Figure 3A–C), while upregulated the expression and transcription of Autophagy Related 5 (ATG5), ferritin autophagy receptor NCOA4 and dynamin-related protein 1 (Drp1), a key regulator of mitochondrial fission, indicating that the potential risk of cell death caused by iron ion was occurring (Figure 3A,D,F).

### 3.3. The Possibility of Ferroptosis Under Conditions That Reduce the Ability of the Virus to Bind to Cells

**Figure 4:**
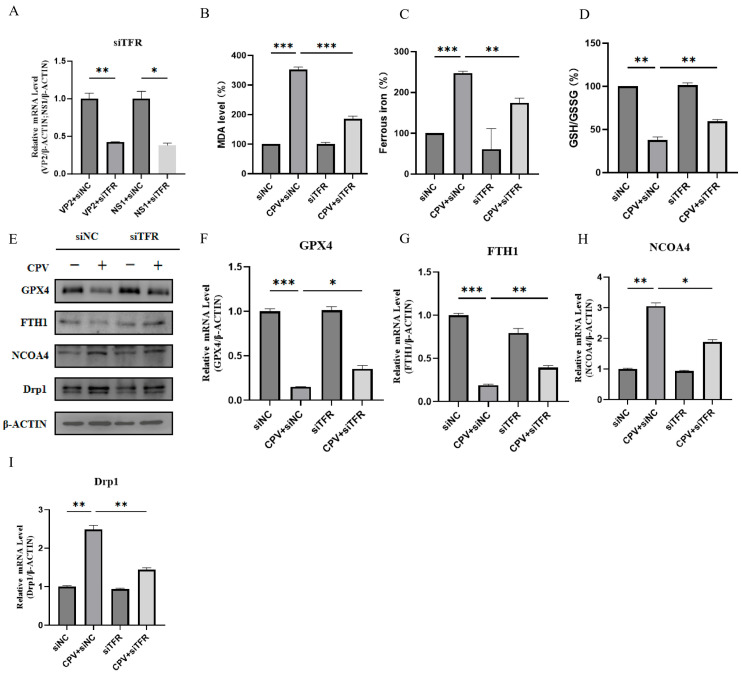
Silencing TFR inhibited viral replication and prevented its induced ferroptosis in CRFK cells. (**A**) qPCR was used to measure VP2 and NS1 mRNA levels in mock- and CPV-infected cells 48 h after siTFR or siNC transfection. (**B**) Lipid peroxidation levels in mock- and virus-infected cells, following transfection with siTFR or siNC, were assessed by measuring malondialdehyde (MDA) concentration in cell lysates using a commercial kit, with absorbance measured at 535 nm. (**C**) The ferrous iron concentration in cell lysates was quantified at 593 nm using a commercial iron assay kit following transfection with siTFR or siNC and subsequent mock infection or viral infection. (**D**) The intracellular GSH/GSSG ratio was determined at 412 nm using a commercial assay kit to assess the redox status in mock-infected and virus-infected cells following transfection with siTFR or siNC. (**E**) Western Blot analysis of GPX4, FTH1, NCOA4, and Drp1 proteins in mock-infected, virus-infected cells with or without the transfection of siTFR or siNC at 48 h. (**F**–**I**) The transcript levels of GPX4, FTH1, NCOA4, and Drp1 mRNA in mock-infected and virus-infected cells at 48 h after siTFR or siNC transfection were measured by qPCR. Data are presented as mean ± SD from *n* = 3 independent experiments; * *p* < 0.05, ** *p* < 0.01, *** *p* < 0.001.

Previous studies have shown that TFR is the receptor for CPV entry into cells. To reduce CPV’s binding to cells, we silenced TFR transcription and then assessed ferroptosis. The results showed that silencing TFR significantly inhibited viral genome replication (Figure 4A) and weakened the ferroptosis phenotype, as indicated by reduced MDA accumulation (Figure 4B), decreased intracellular ferrous iron levels (Figure 4C), and upregulation of the GSH/GSSG ratio (Figure 4D). At the molecular level, silencing of TFR resulted in upregulation of GPX4 and FTH1 and inhibition of NCOA4 and Drp1 (Figure 4E–I). These findings provide further strong evidence that CPV induces ferroptosis in host cells.

### 3.4. The Iron Chelating Agent DFO Mitigates CPV-Induced Ferroptosis and Suppresses Viral Genome Replication

**Figure 5:**
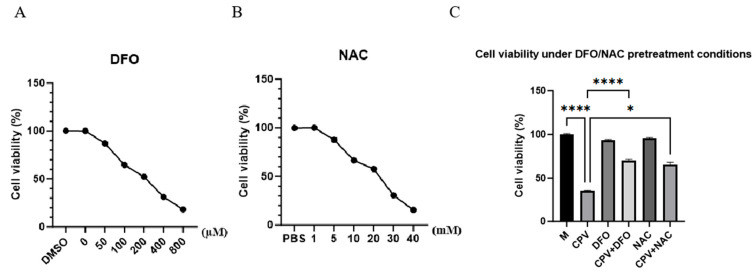
CCK-8 assays confirmed that DFO (50 μM) and NAC (5 mM) were non-cytotoxic and improved CPV-reduced viability. (**A**) Cell viability after treatment with different concentrations of DFO. (**B**) Cell viability after treatment with different concentrations of NAC. (**C**) DFO (50 μM) and NAC (5 mM) showed no cytotoxicity and partially rescued CPV-induced loss of cell viability in CRFK cells. Data are presented as mean ± SD from *n* = 3 independent experiments; * *p* < 0.05, **** *p* < 0.0001.

To verify the potential role of DFO, CRFK cells were pretreated with the iron chelator DFO (50 μM) or the antioxidant N-acetylcysteine (NAC, 5 mM) prior to CPV inoculation (Figure 5A,B). These concentrations were chosen based on CCK-8 viability assays, and neither DFO nor NAC produced significant cytotoxicity under the same pretreatment–infection schedule used for the replication analyses (Figure 5C), thereby excluding nonspecific toxic pre-exposure as an explanation for reduced viral replication. NAC is widely used in antiviral and ferroptosis-related studies as a reference antioxidant that lowers ROS and replenishes intracellular cysteine and glutathione pools, although it is not a specific ferroptosis inhibitor. Accordingly, including NAC allowed us to distinguish whether CPV-induced cellular injury results primarily from excessive ROS or from iron-dependent lipid peroxidation characteristic of ferroptosis.

**Figure 6:**
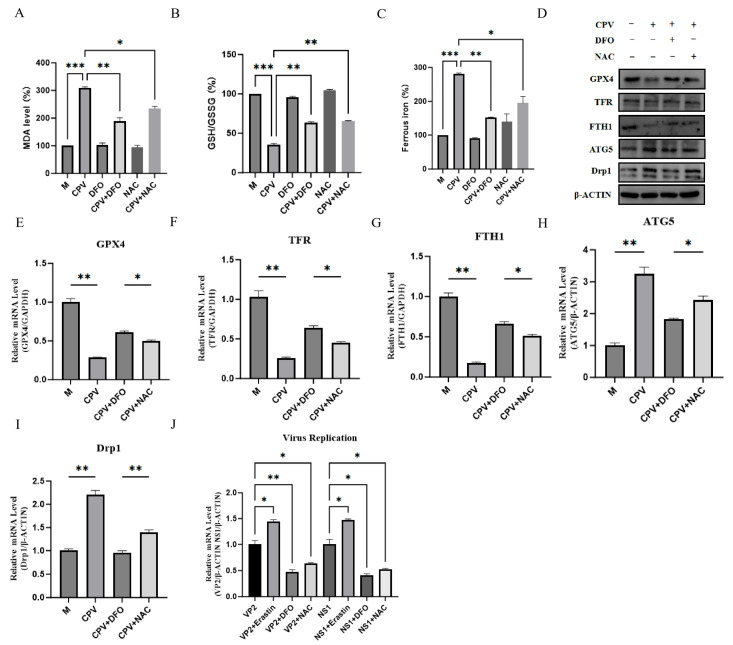
DFO attenuates canine parvovirus replication and virus-induced host cell ferroptosis. (**A**) To evaluate lipid peroxidation, MDA concentrations in cell lysates were determined at 535 nm. Measurements were taken from mock-infected and virus-infected cells that had been pretreated with either DFO or NAC, respectively. (**B**) The intracellular GSH/GSSG ratio was determined at 412 nm using a commercial assay kit to assess the redox state in mock-infected or virus-infected cells following treatment with DFO or NAC. (**C**) The ferrous iron concentration in cell lysates was quantified using a commercial iron assay kit at 593 nm following treatment with DFO or NAC and subsequent mock or viral infection. (**D**) The protein expression levels of GPX4, FTH1, NCOA4, and Drp1 were analyzed by Western Blot in mock-infected and virus-infected cells following treatment with DFO. (**E**–**I**) The mRNA expression levels of GPX4, FTH1, NCOA4, and Drp1 were determined by qPCR in mock-infected and CPV-infected cells following DFO treatment. (**J**) qPCR was used to detect the transcriptional levels of VP2 and NS1 mRNA in simulated and virus-infected cells with or without Erastin pretreatment and subsequent DFO or NAC treatment. Data are presented as mean ± SD from n = 3 independent experiments; * *p* < 0.05, ** *p* < 0.01, *** *p* < 0.001.

The results showed that DFO could significantly alleviate ferroptosis induced by CPV, specifically manifested as a reduction in the accumulation of lipid peroxidation product MDA (Figure 6A), an upregulation of the GSH/GSSG ratio (Figure 6B), and a decrease in intracellular ferrous ion levels (Figure 6C). Western Blot and qPCR analyses further demonstrated that DFO treatment reversed the downregulation of GPX4, TFR, and FTH1 at the protein and mRNA levels caused by CPV infection, and inhibited the abnormally high expression of ATG5 and Drp1 (Figure 6D–I). More importantly, DFO significantly suppressed CPV replication, as indicated by reduced VP2 and NS1 transcription compared with CPV infection alone. In contrast, erastin pretreatment, used to induce ferroptosis, markedly enhanced VP2 and NS1 mRNA levels, whereas both DFO and NAC attenuated this ferroptosis-associated increase in viral replication, with DFO showing a stronger inhibitory effect than NAC (Figure 6J). The working concentration of Erastin was selected based on CCK-8 viability assays, which confirmed no significant cytotoxicity under our experimental conditions (Figure S4).

## 4. Discussion

Ferroptosis, an iron-dependent form of regulated cell death, is widely implicated in diverse pathological processes, including neurodegeneration, ischemia/reperfusion injury, and tumor suppression [32,33]. Increasing evidence indicates that viruses can differentially modulate ferroptotic pathways through distinct molecular mechanisms, thereby shaping the course and outcome of infection [34]. For example, herpes simplex virus type 1 (HSV-1) induces ferroptosis by degrading the antioxidant transcription factor Nrf2, disrupting cellular redox homeostasis, and exacerbating viral encephalitis [35]. Swine influenza virus (SIV) infection suppresses the expression of GPX4, a key defense protein against ferroptosis, leading to glutathione depletion and lipid peroxide accumulation, which not only triggers ferroptosis but also facilitates viral replication [36]. NDV infection decreases NCOA4 and FTH1, resulting in ferrous release and lipid ROS accumulation that culminates in ferroptosis [37,38].

In our study, CPV infection induced a dose-dependent ferroptosis-like phenotype in CRFK cells. This was evidenced by increased lipid peroxidation and iron stress, impaired glutathione-dependent antioxidant capacity, elevated oxidative load, coordinated GPX4 downregulation and ACSL4 upregulation, and ferroptosis-associated mitochondrial injury. Moreover, TFR silencing reduced viral replication and rescued ferroptosis-related readouts, supporting a model in which receptor-mediated infection drives iron dysregulation and ferroptosis induction. Together with our gain- and loss-of-function data showing that ferroptosis inhibition (DFO/NAC) suppresses CPV replication, whereas ferroptosis induction (Erastin) enhances VP2/NS1 expression, ferroptotic stress appears to functionally promote CPV propagation rather than merely accompany infection. These observations underscore the recurring involvement of ferroptosis and iron-regulatory proteins in viral pathogenesis.

Collectively, these findings indicate that viral interference with ferroptotic signaling is closely linked to manipulation of host iron metabolism [39,40]. Disruption of iron homeostasis not only accelerates lipid peroxidation and oxidative injury but also establishes a microenvironment conducive to viral replication. In this context, maintaining intracellular iron balance emerges as a key factor influencing both viral propagation and host cell survival [41,42]. Thus, pharmacological modulation of iron availability may represent an effective approach to counter virus-induced ferroptosis [43].

DFO was approved for clinical use in the United States in 1968 and is included in the World Health Organization Essential Medicines List. Its clinical use in managing iron overload disorders, such as β-thalassemia, demonstrates a well-characterized safety profile and extensive pharmacological data [44]. However, its application in veterinary medicine remains limited. The potential value of DFO in treating CPV infection is of particular interest, given that CPV relies on TFR for cellular entry and disrupts iron homeostasis to promote replication and ferroptosis [45].

A notable advantage of DFO lies in its host-directed mechanism of action. Although DFO does not directly bind or inhibit TFR, it effectively reduces the intracellular labile iron pool by chelating ferric ions, thereby indirectly modulating TFR-regulated pathways. Because CPV replication depends on iron availability and ferroptotic signaling, DFO-mediated reduction of intracellular Fe^3^⁺/Fe^2^⁺ interrupts iron-dependent processes required for efficient viral propagation. Since all known CPV variants utilize TFR for entry and rely on iron dysregulation to establish a replication-permissive environment, DFO can alleviate virus-induced disturbances in iron metabolism and thereby suppress CPV replication [46]. Our experimental results confirmed that DFO treatment significantly inhibited CPV replication and attenuated the ferroptosis response.

An important observation from this study is that CPV infection leads to a downregulation of TFR during the later stages of infection. Although CPV uses TFR for cellular entry, many viruses—including influenza virus—reduce surface levels of their entry receptors after internalization as a superinfection-exclusion strategy, thereby limiting secondary infections and preventing additional virions from competing for cellular resources [47,48]. Furthermore, reduced TFR expression may contribute to intracellular iron dysregulation by limiting iron uptake, while ferritin degradation and impaired iron export increase the labile Fe^2+^ pool [49,50]. Such alterations promote ROS generation and enhance cellular susceptibility to ferroptosis, which may be beneficial for CPV replication. Thus, while TFR is essential for CPV entry, its subsequent downregulation likely reflects a viral strategy to manipulate host iron metabolism and maintain a ferroptosis-prone cellular environment that supports efficient viral propagation [51].

Although our in vitro findings provide mechanistic insight into the interplay among iron metabolism, ferroptosis, and CPV replication, further in vivo studies are required to fully evaluate the therapeutic potential of DFO. Key parameters, including pharmacokinetics, tissue distribution, optimal dosing regimens, efficacy, and safety, should be systematically assessed in canine models [52]. Nevertheless, given its long-standing clinical approval and well-characterized pharmacological profile, DFO represents a promising candidate for repurposing as a host-directed antiviral agent. Overall, our study expands the understanding of CPV pathogenesis and supports ferroptosis inhibition via iron chelation as a potential therapeutic strategy in veterinary medicine [53].

## 5. Conclusions

**Figure 7:**
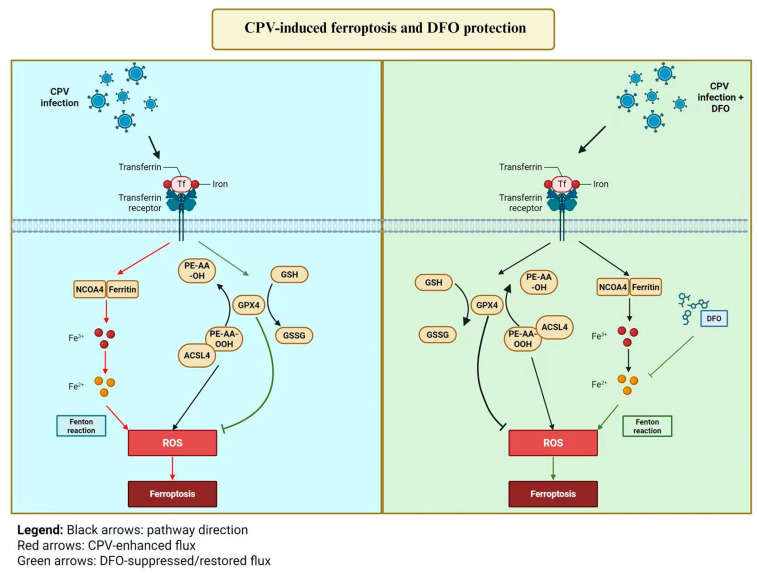
Proposed model of CPV-induced ferroptosis and DFO protection in CRFK cells. CPV infection disrupts intracellular iron homeostasis, increases ROS production, and triggers ferroptosis-associated changes, including lipid peroxidation, mitochondrial dysfunction, and altered expression of GPX4, ACSL4, TFR, and NCOA4. DFO chelates intracellular iron and alleviates ferroptosis, thereby suppressing viral replication.

This study identifies ferroptosis as an important pathogenic component of CPV infection. DFO mitigates CPV-induced ferroptotic stress by chelating intracellular iron, which in turn alleviates lipid peroxidation and significantly suppresses viral replication, highlighting DFO as a promising host-directed candidate for CPV therapy (Figure 7). Proposed model of CPV-induced ferroptosis and DFO protection in CRFK cells.

## Data Availability

The original contributions presented in this study are included in the article/Supplementary Materials. Further inquiries can be directed to the corresponding authors.

## Supplementary Materials

The following supporting information can be downloaded at https://www.mdpi.com/article/10.3390/vetsci12121192/s1; Supplementary Methods S1: Calculation of MDA content. Supplementary Methods S2: Calculation of GSH/GSSG ratio. Figure S1: Dose-dependent effects of CPV infection on ferroptosis and iron-metabolism–related gene expression in CRFK cells. Figure S2: Silencing TFR inhibited viral replication and prevented its induction of ferroptosis in CRFK cells. Figure S3: DFO attenuates canine parvovirus replication and virus-induced host cell ferroptosis. Figure S4: Dose–response analysis of erastin cytotoxicity in CRFK cells by CCK-8 assay. Figure S5: Quantification of western blot band intensities. Table S1: The primers used in this study.
