# Supplementary material for: Deferoxamine Inhibits Canine Parvovirus by Suppressing Ferroptosis and Viral Replication
Source: Vet Sci. 2025 Dec 12;12(12):1192. doi: 10.3390/vetsci12121192 (PMC12737514; doi:10.3390/vetsci12121192)
Supplement: Supplementary file 1 [file vetsci-12-01192-s001.zip › Figure S2.pdf]

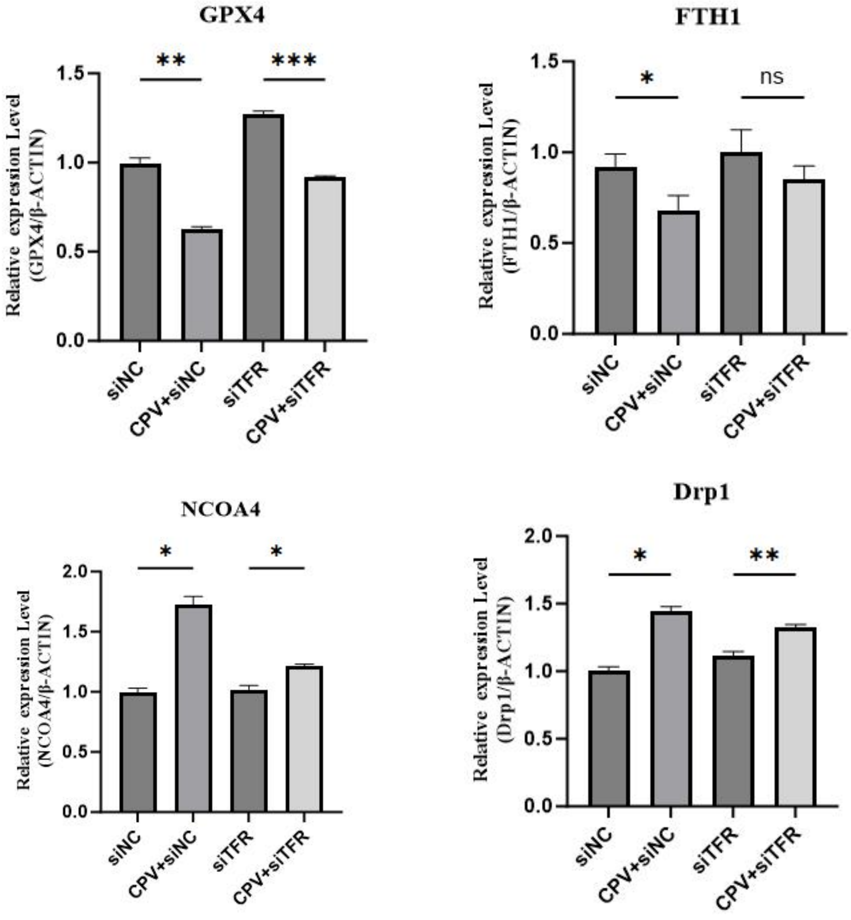

Figure S2. Silencing TFR inhibited viral replication and inhibited its induced ferroptosis in CRFK cells.
