# Supplementary material for: Deferoxamine Inhibits Canine Parvovirus by Suppressing Ferroptosis and Viral Replication
Source: Vet Sci. 2025 Dec 12;12(12):1192. doi: 10.3390/vetsci12121192 (PMC12737514; doi:10.3390/vetsci12121192)
Supplement: Supplementary file 1 [file vetsci-12-01192-s001.zip › Figure S5.pdf]

Figure S5 (1).(E) Western Blot was used to detect the protein expression of GPX4 and ACSL4 in cells under different MOIs.

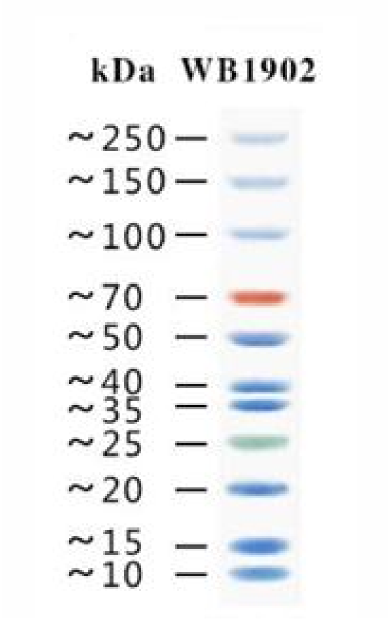

boitides WB1902

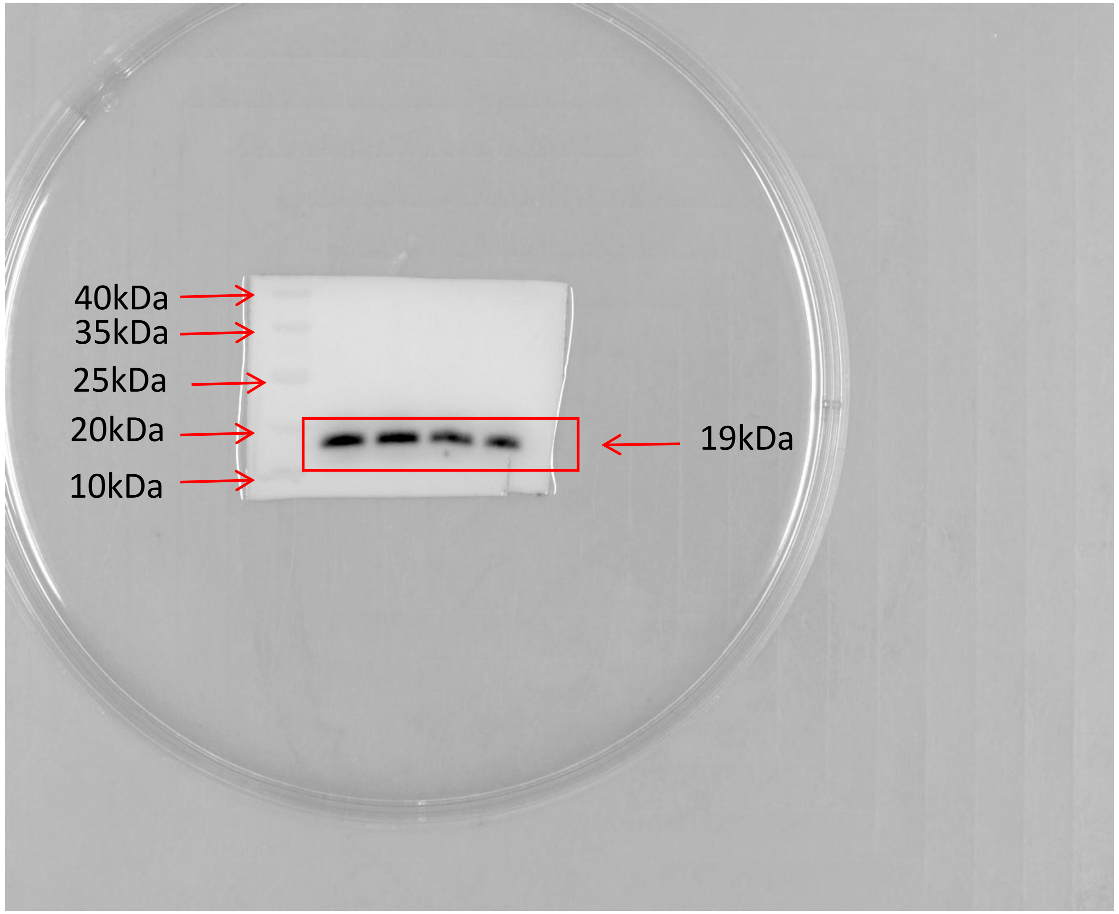

GPX4

Figure S5 (1).(E) Western Blot was used to detect the protein expression of GPX4 and ACSL4 in cells under different MOIs.

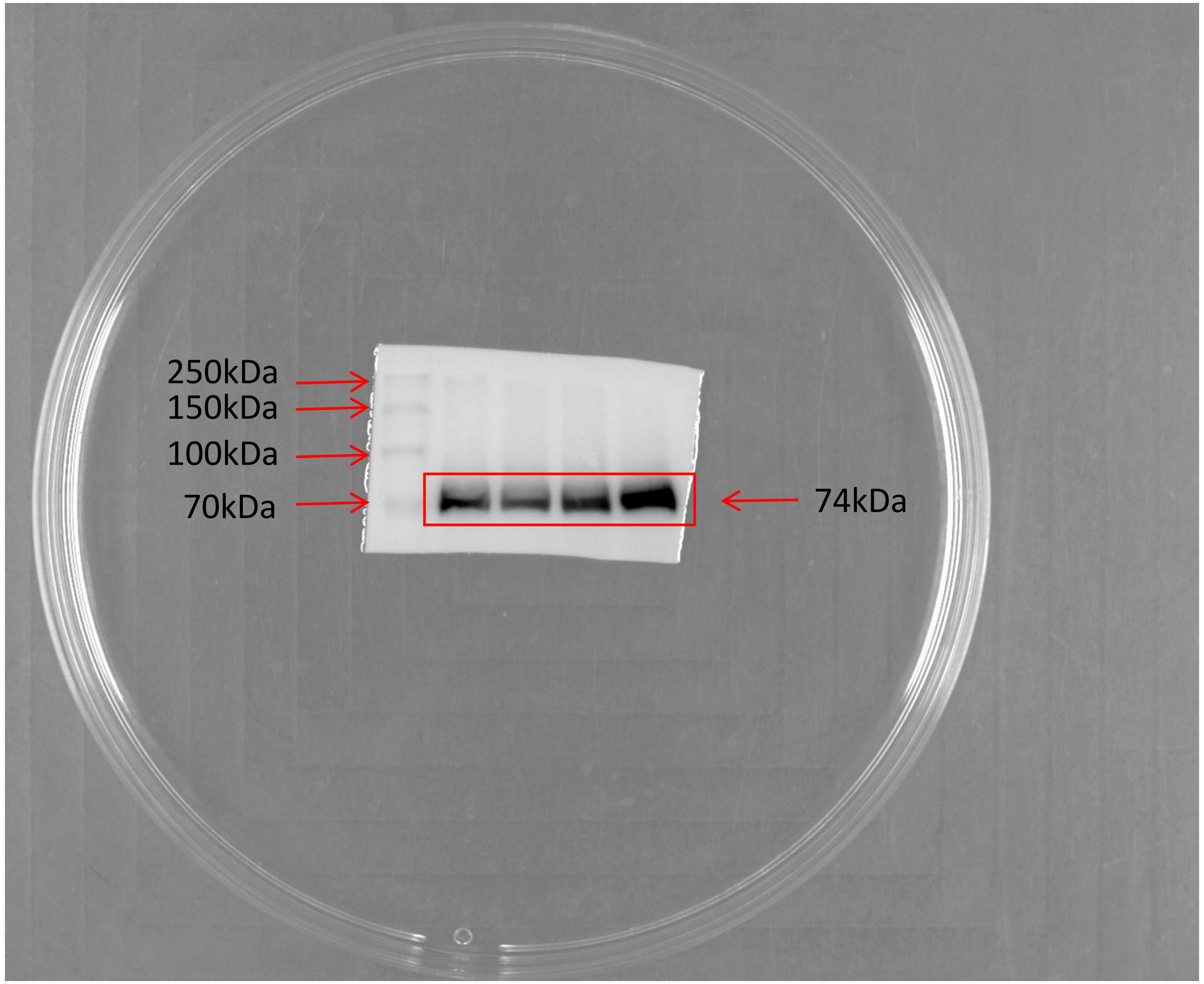

ACSL4

Figure S5 (1).(E) Western Blot was used to detect the protein expression of GPX4 and ACSL4 in cells under different MOIs.

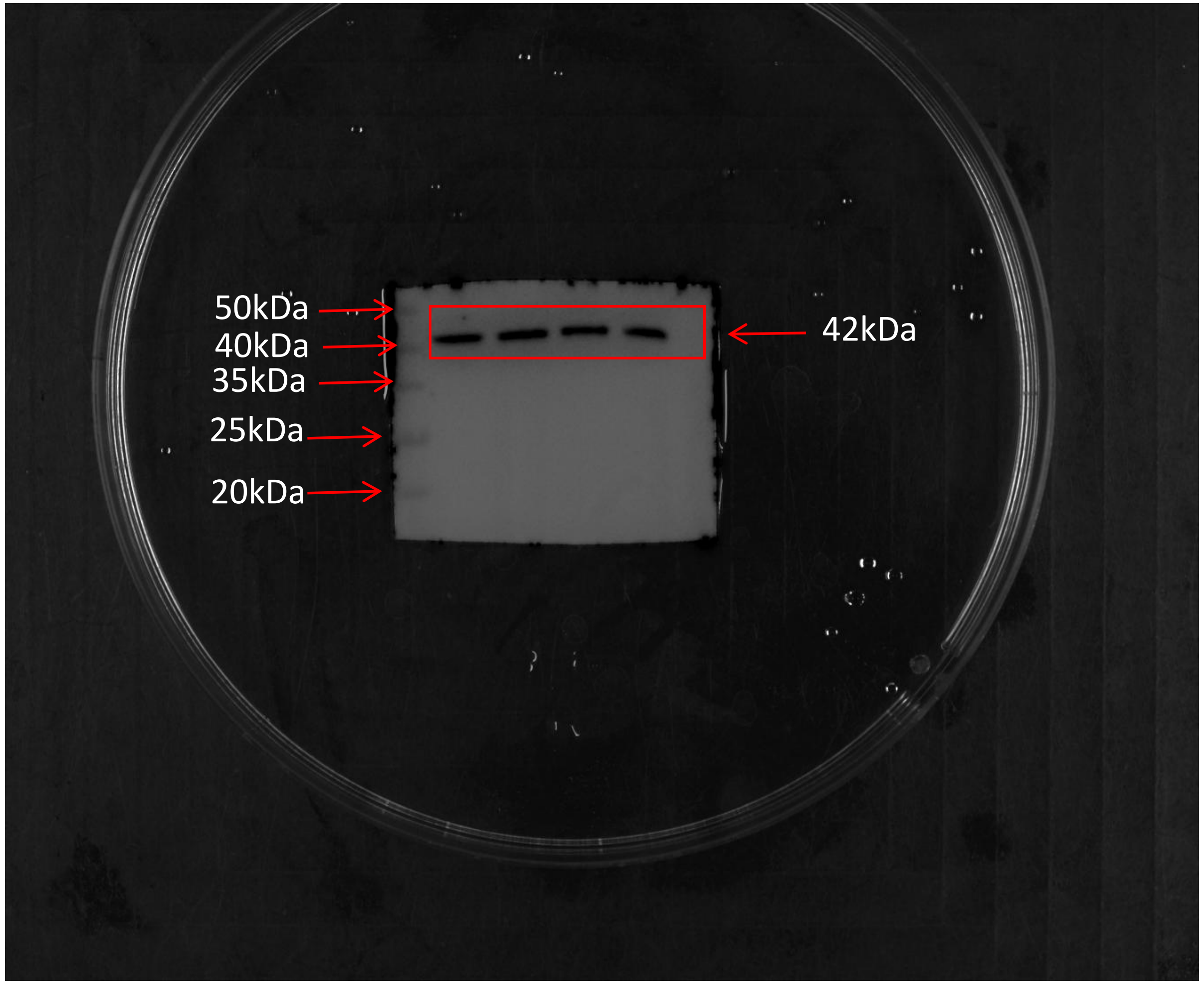

β-ACTIN

Figure S5 (2).(C) Western Blot was used to detect the expression of TFR, FTH1, ATG5, NCOA4 and Drp1 in cells under different MOIs.

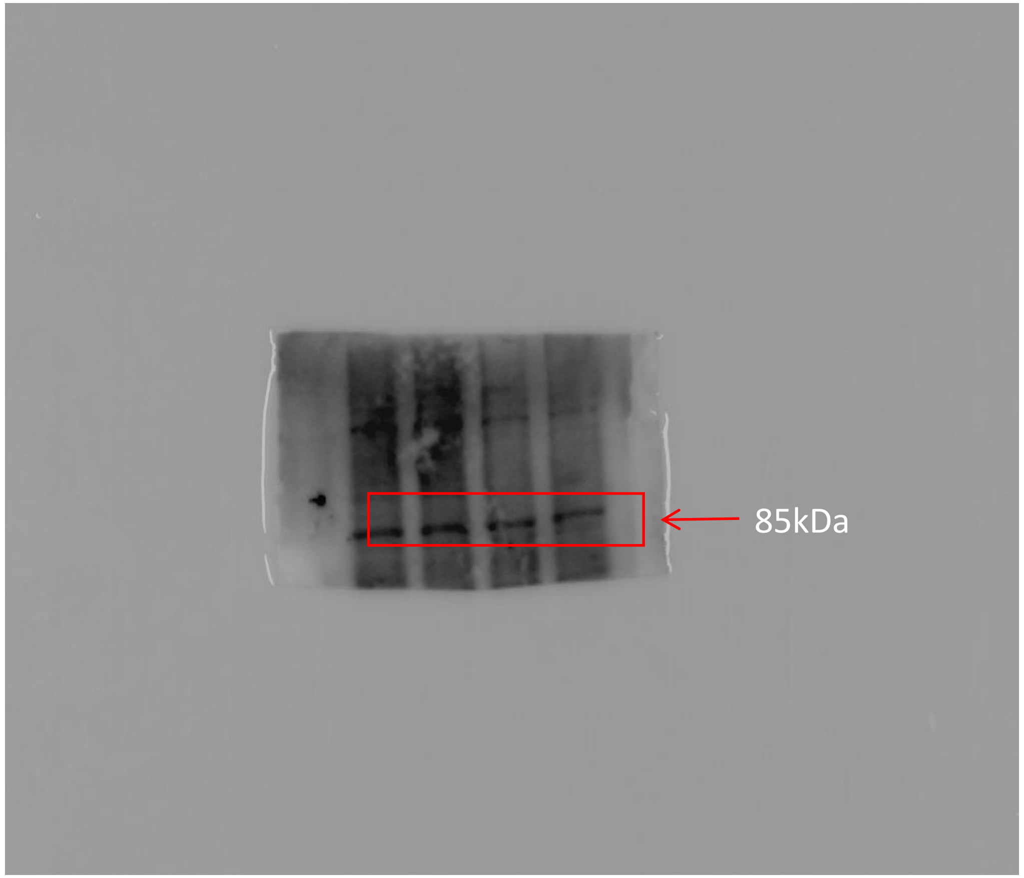

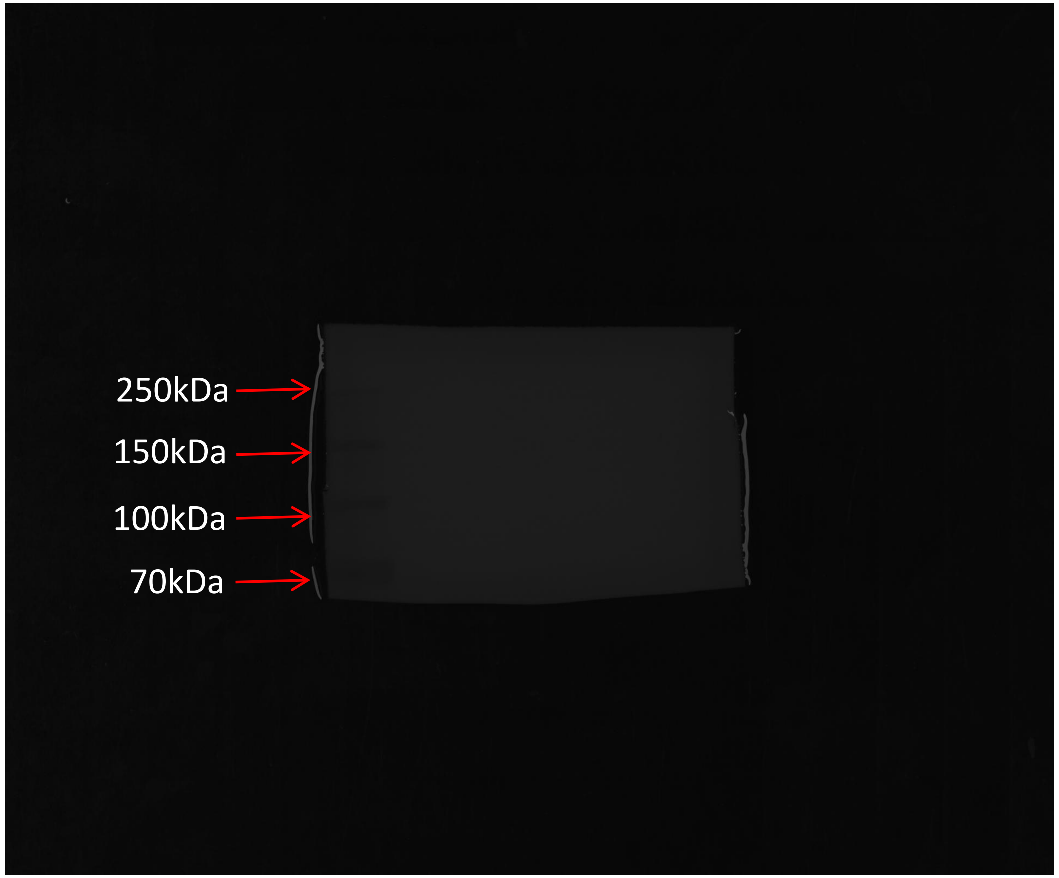

TFR

Figure S5 (2).(C) Western Blot was used to detect the expression of TFR, FTH1, ATG5, NCOA4 and Drp1 in cells under different MOIs.

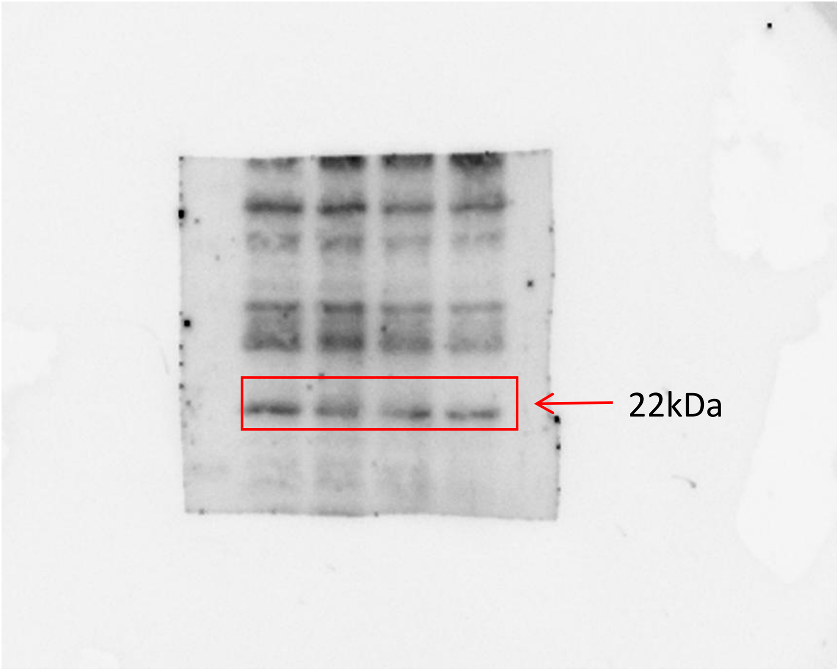

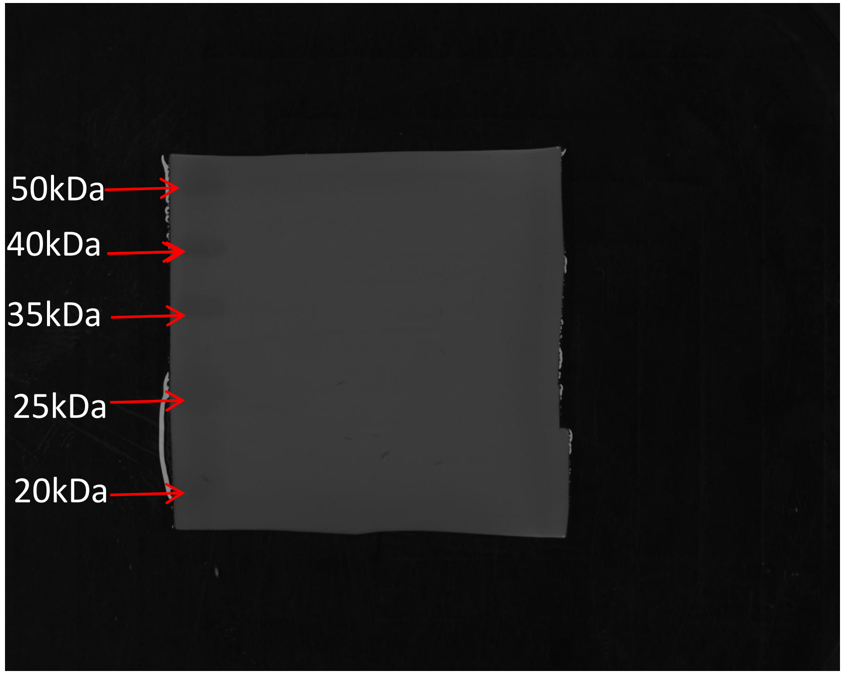

FTH1

Figure S5 (2).(C) Western Blot was used to detect the expression of TFR, FTH1, ATG5, NCOA4 and Drp1 in cells under different MOIs.

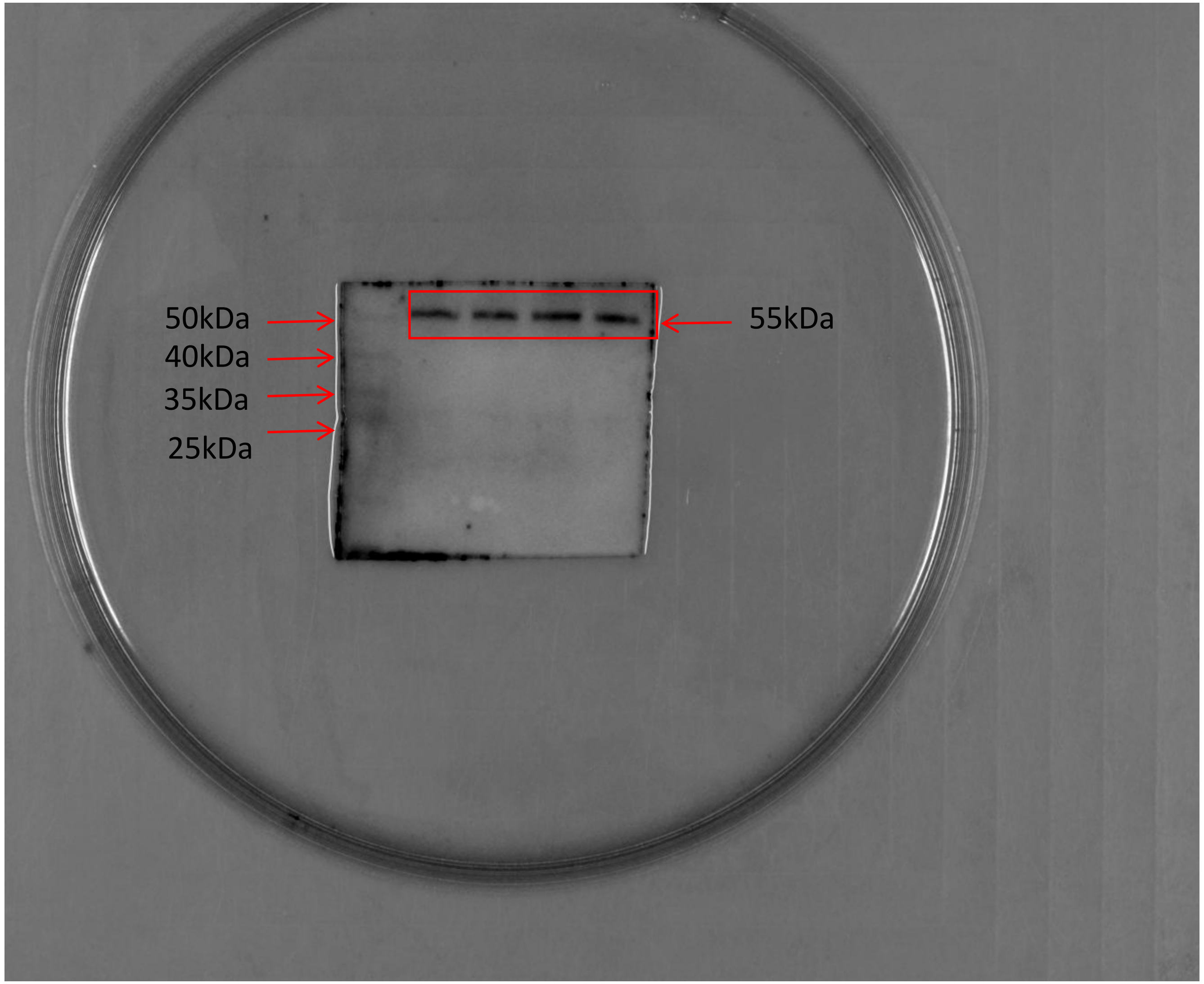

ATG5

Figure S5 (2).(C) Western Blot was used to detect the expression of TFR, FTH1, ATG5, NCOA4 and Drp1 in cells under different MOIs.

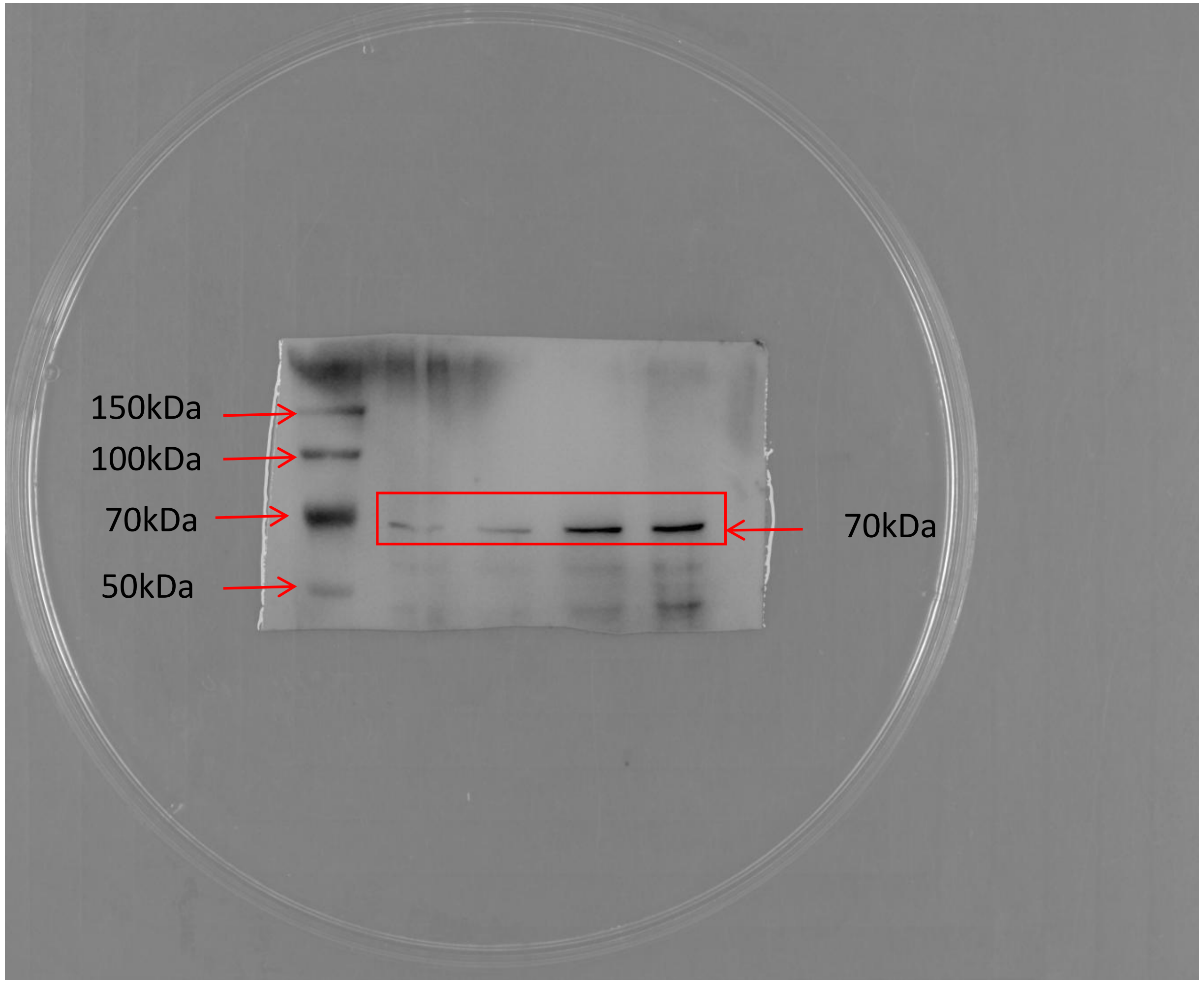

NCOA4

Figure S5 (2).(C) Western Blot was used to detect the expression of TFR, FTH1, ATG5, NCOA4 and Drp1 in cells under different MOIs.

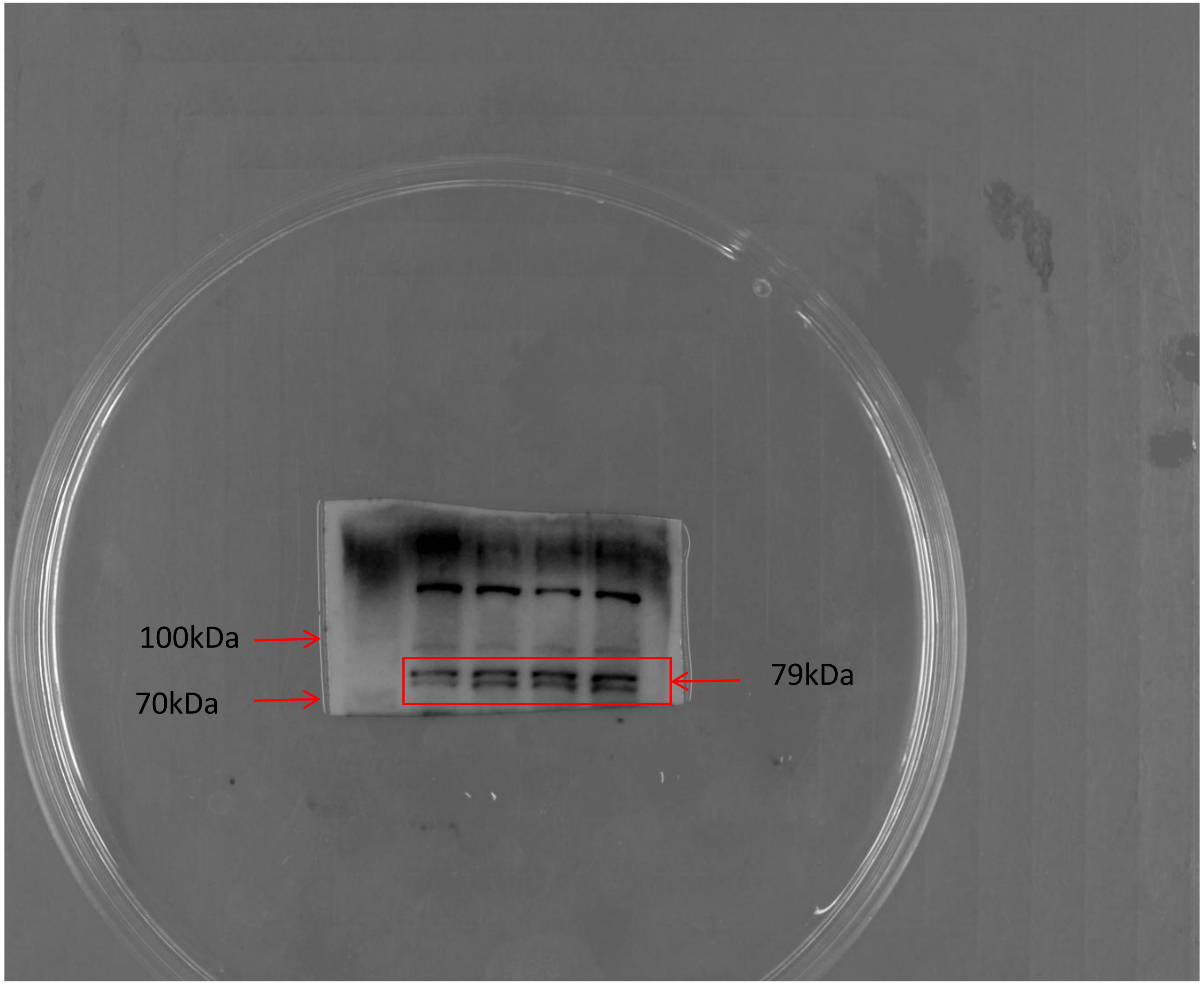

DRP1

Figure S5 (2).(C) Western Blot was used to detect the expression of TFR, FTH1, ATG5, NCOA4 and Drp1 in cells under different MOIs.

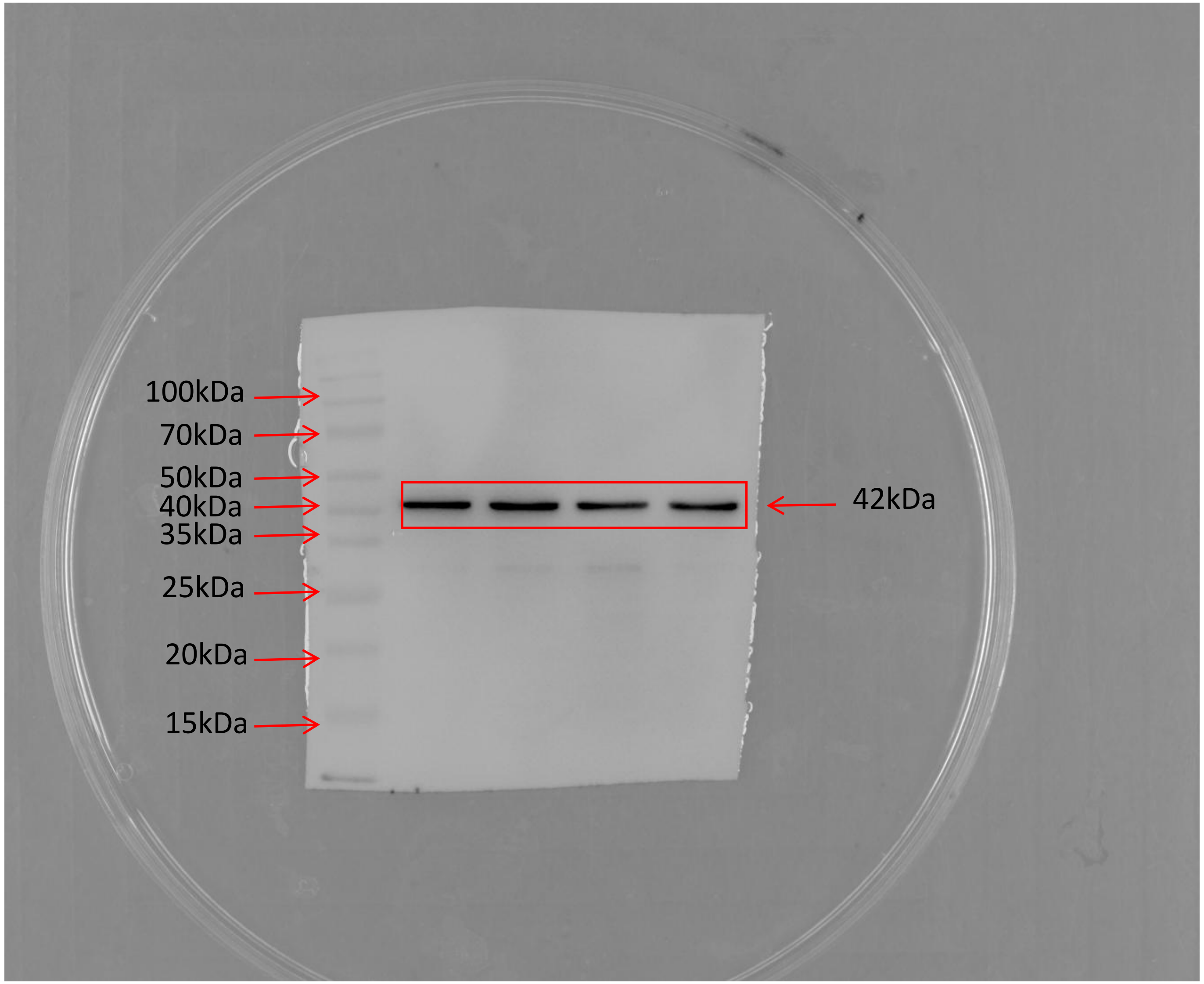

β-ACTIN

Figure S5 (3).(E) Western blot analysis of GPX4、FTH1、NCOA4 and Drp1 proteins in mock-infected, virus-infected cells with or without the transfection of siTFR or siNC at 48 h.

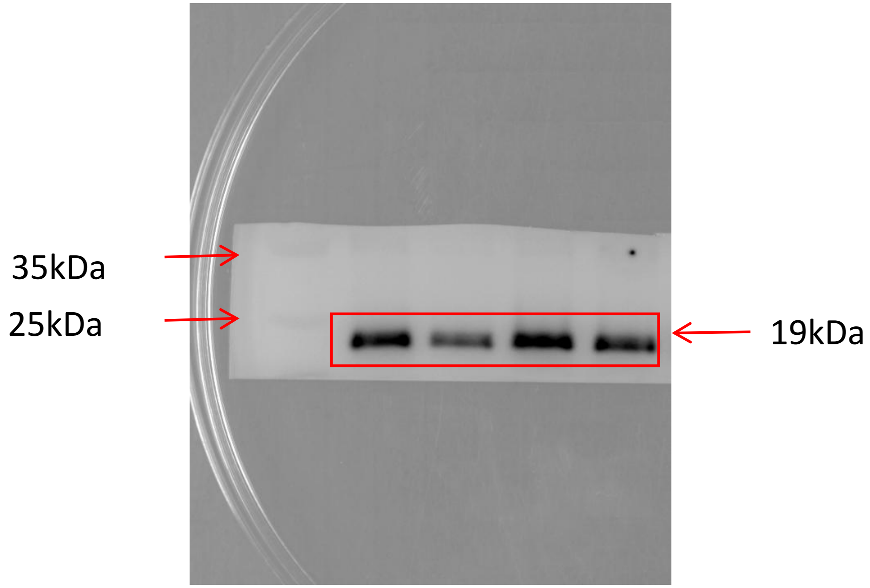

GPX4

Figure S5 (3).(E) Western blot analysis of GPX4、FTH1、NCOA4 and Drp1 proteins in mock-infected, virus-infected cells with or without the transfection of siTFR or siNC at 48 h.

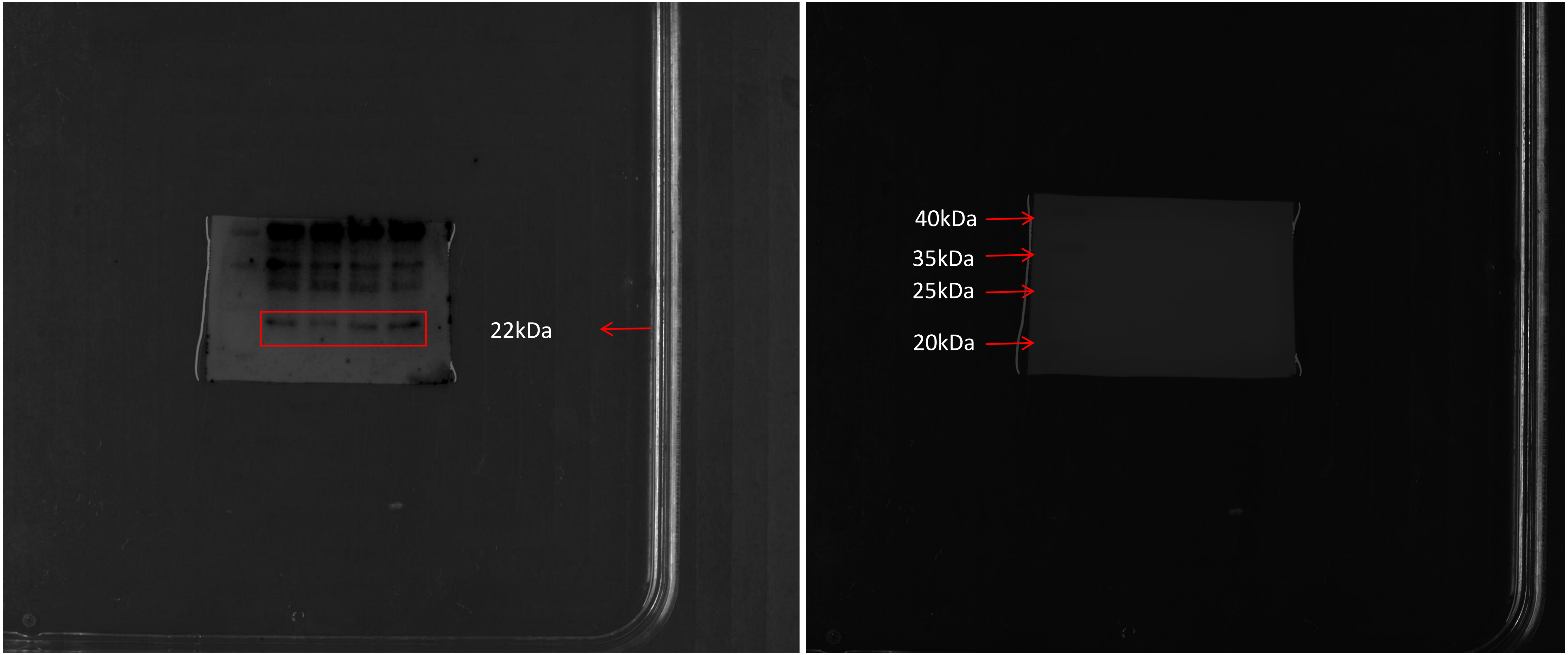

FTH1

Figure S5 (3).(E) Western blot analysis of GPX4、FTH1、NCOA4 and Drp1 proteins in mock-infected, virus-infected cells with or without the transfection of siTFR or siNC at 48 h.

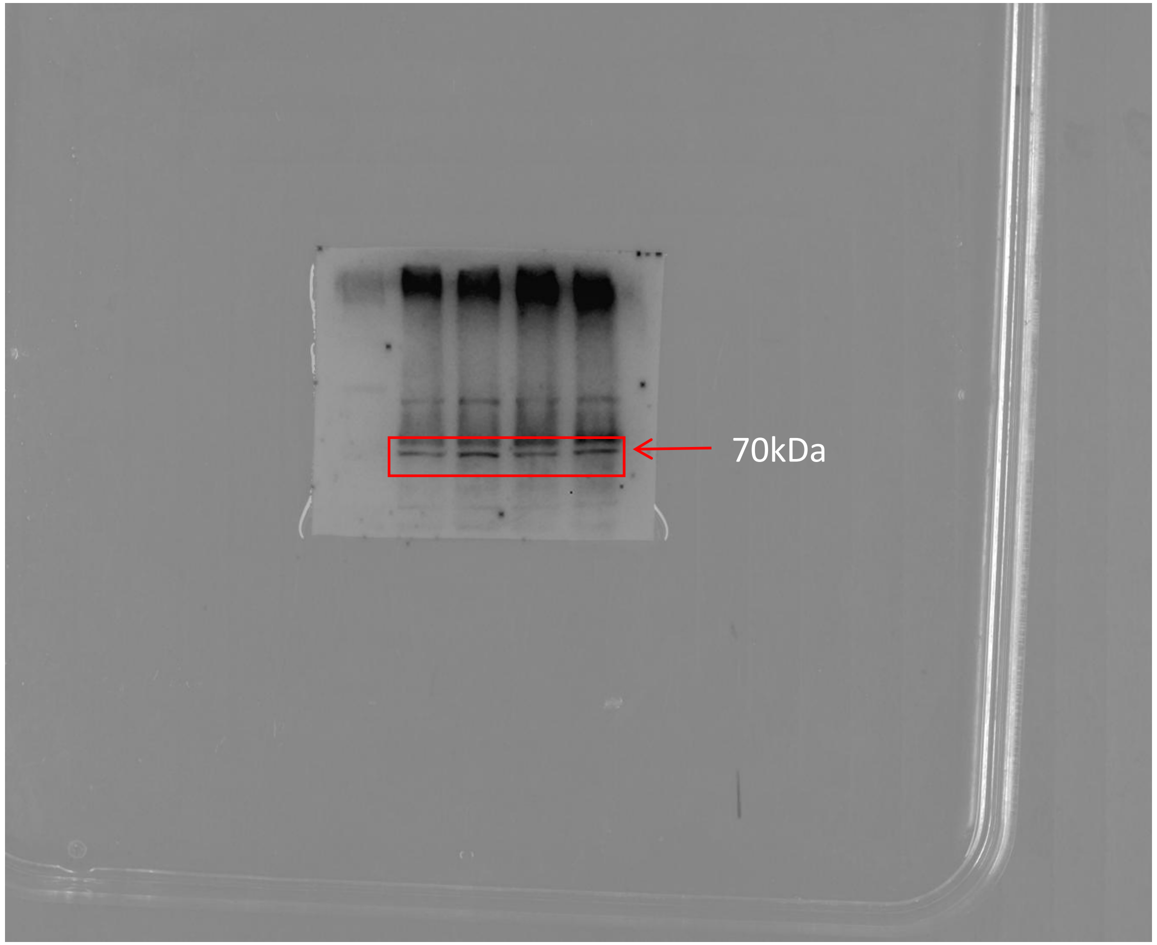

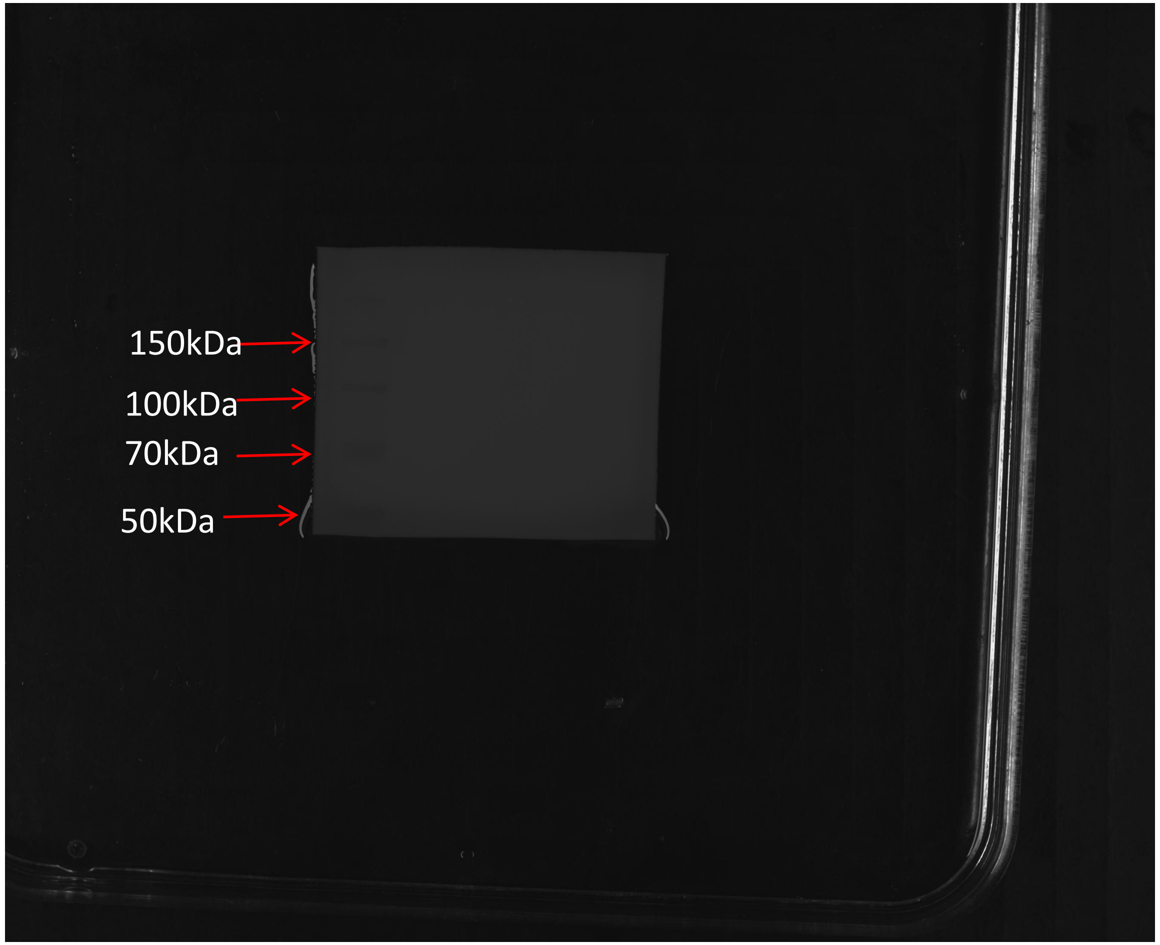

NCOA4

Figure S5 (3).(E) Western blot analysis of GPX4、FTH1、NCOA4 and Drp1 proteins in mock-infected, virus-infected cells with or without the transfection of siTFR or siNC at 48 h.

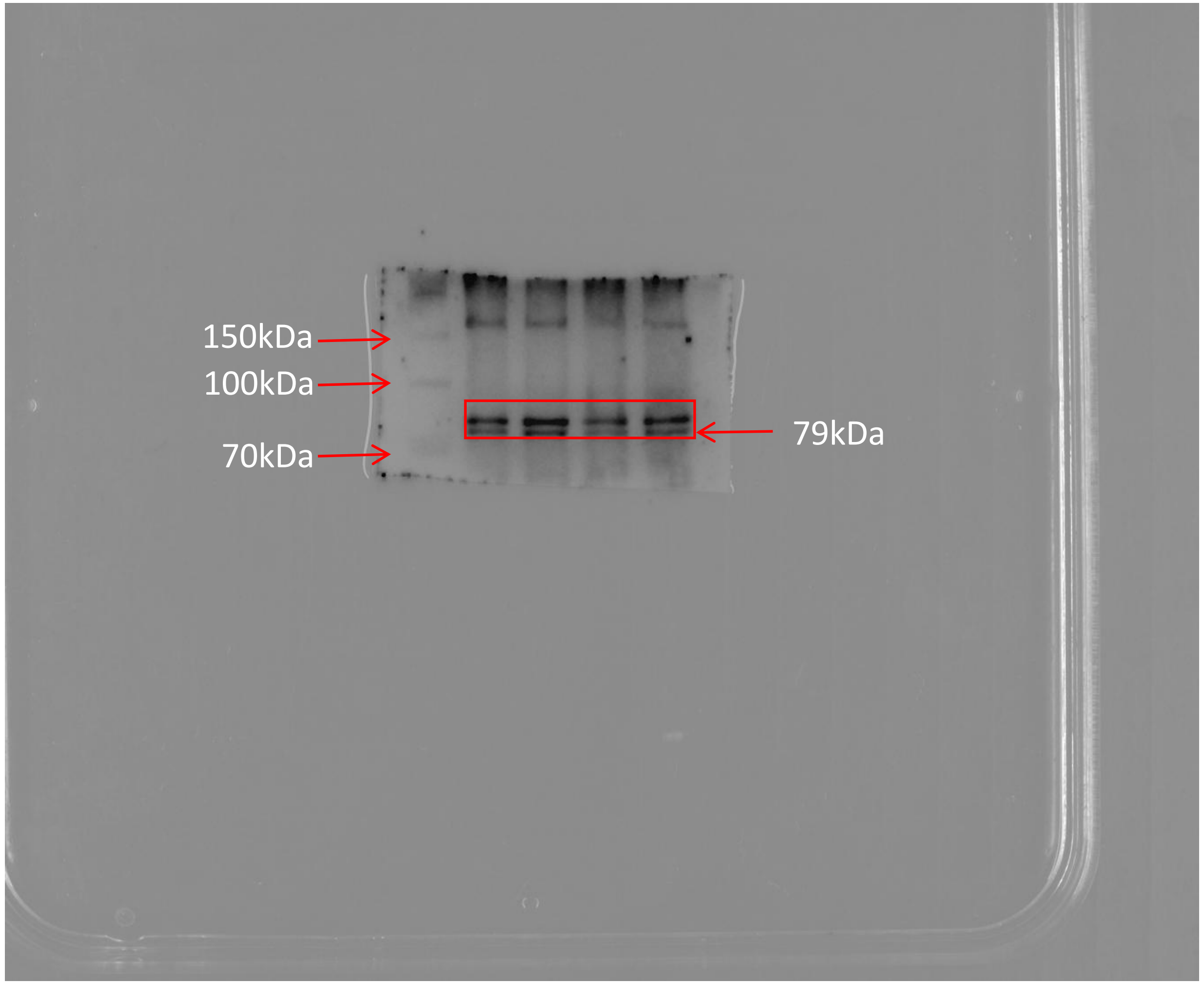

DRP1

Figure S5 (3).(E) Western blot analysis of GPX4、FTH1、NCOA4 and Drp1 proteins in mock-infected, virus-infected cells with or without the transfection of siTFR or siNC at 48 h.

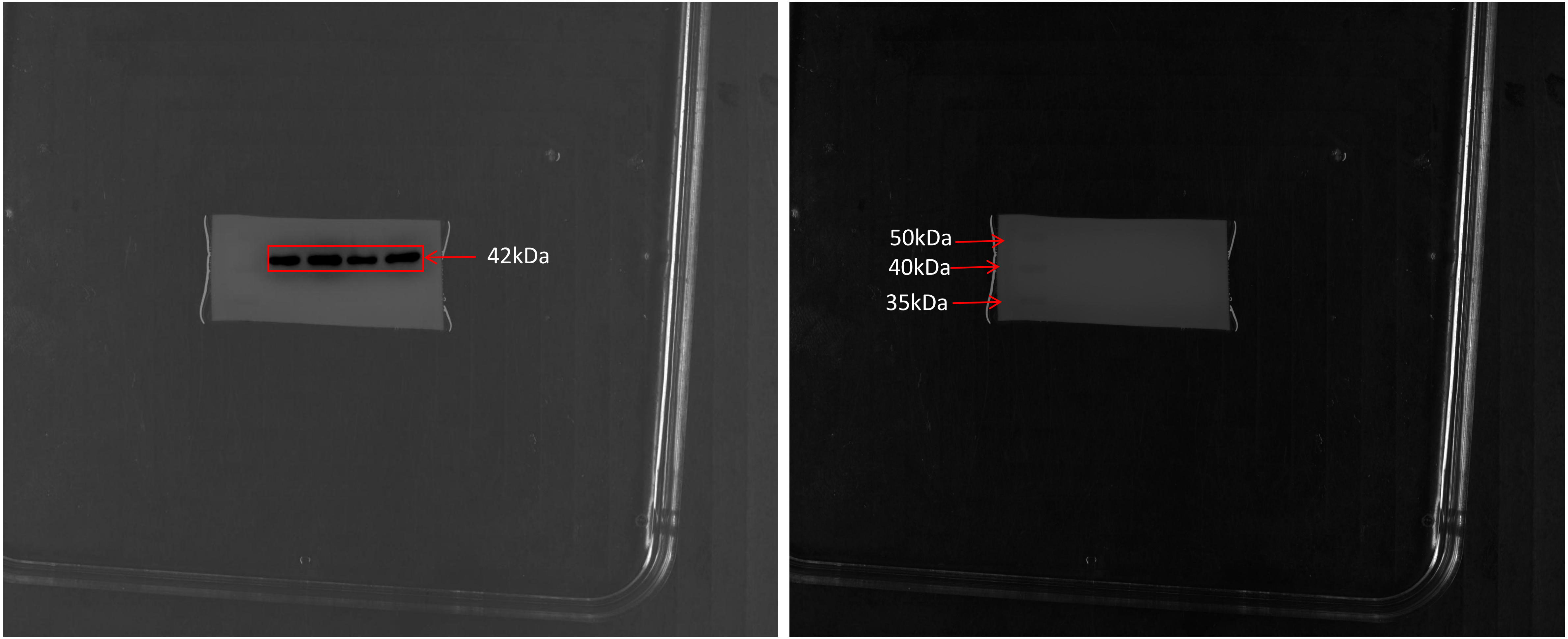

β-ACTIN

Figure S5 (3).(E) Western blot analysis of GPX4、FTH1、NCOA4 and Drp1 proteins in mock-infected, virus-infected cells with or without the transfection of siTFR or siNC at 48 h.

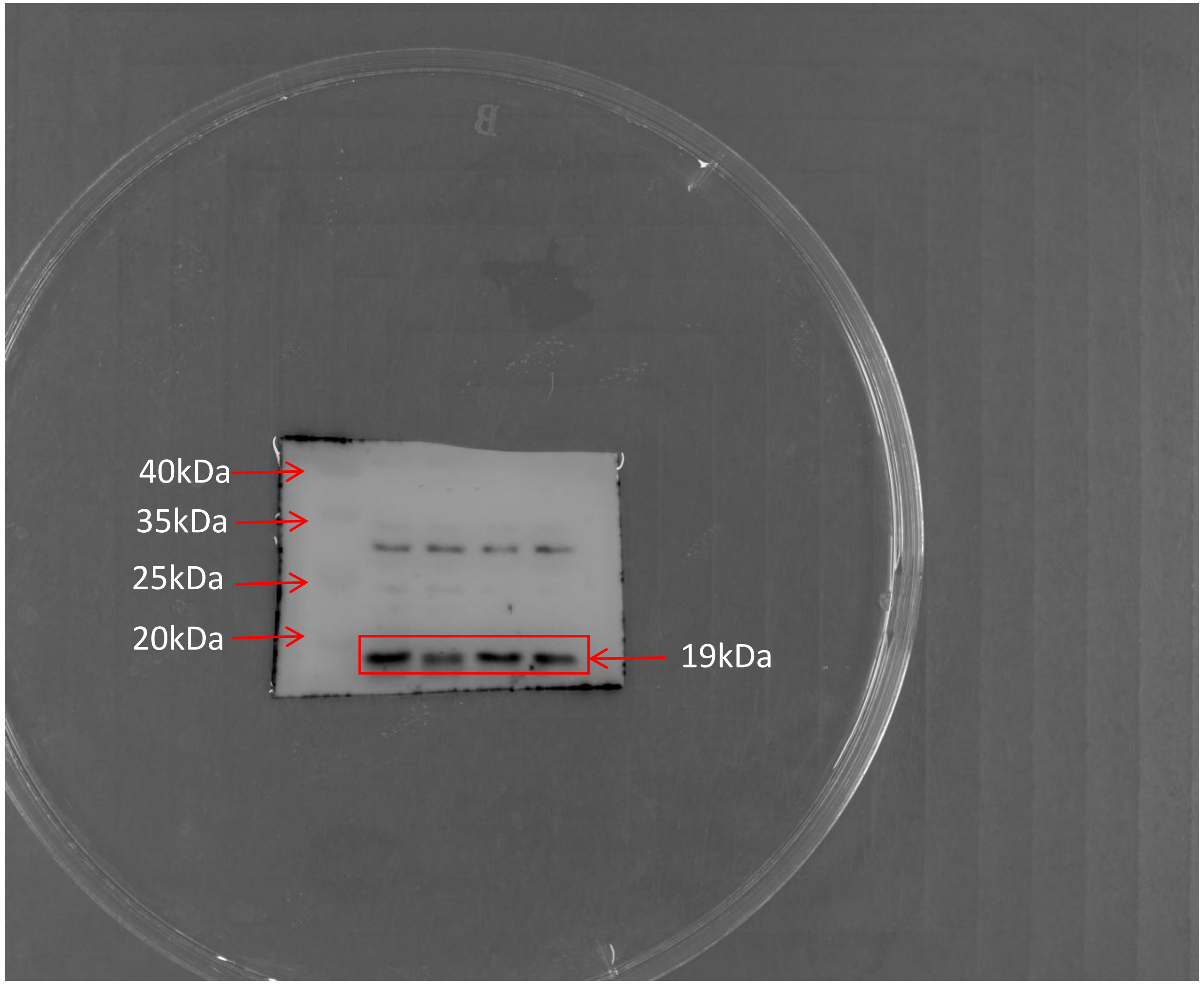

GPX4

Figure S5 (3).(E) Western blot analysis of GPX4、FTH1、NCOA4 and Drp1 proteins in mock-infected, virus-infected cells with or without the transfection of siTFR or siNC at 48 h.

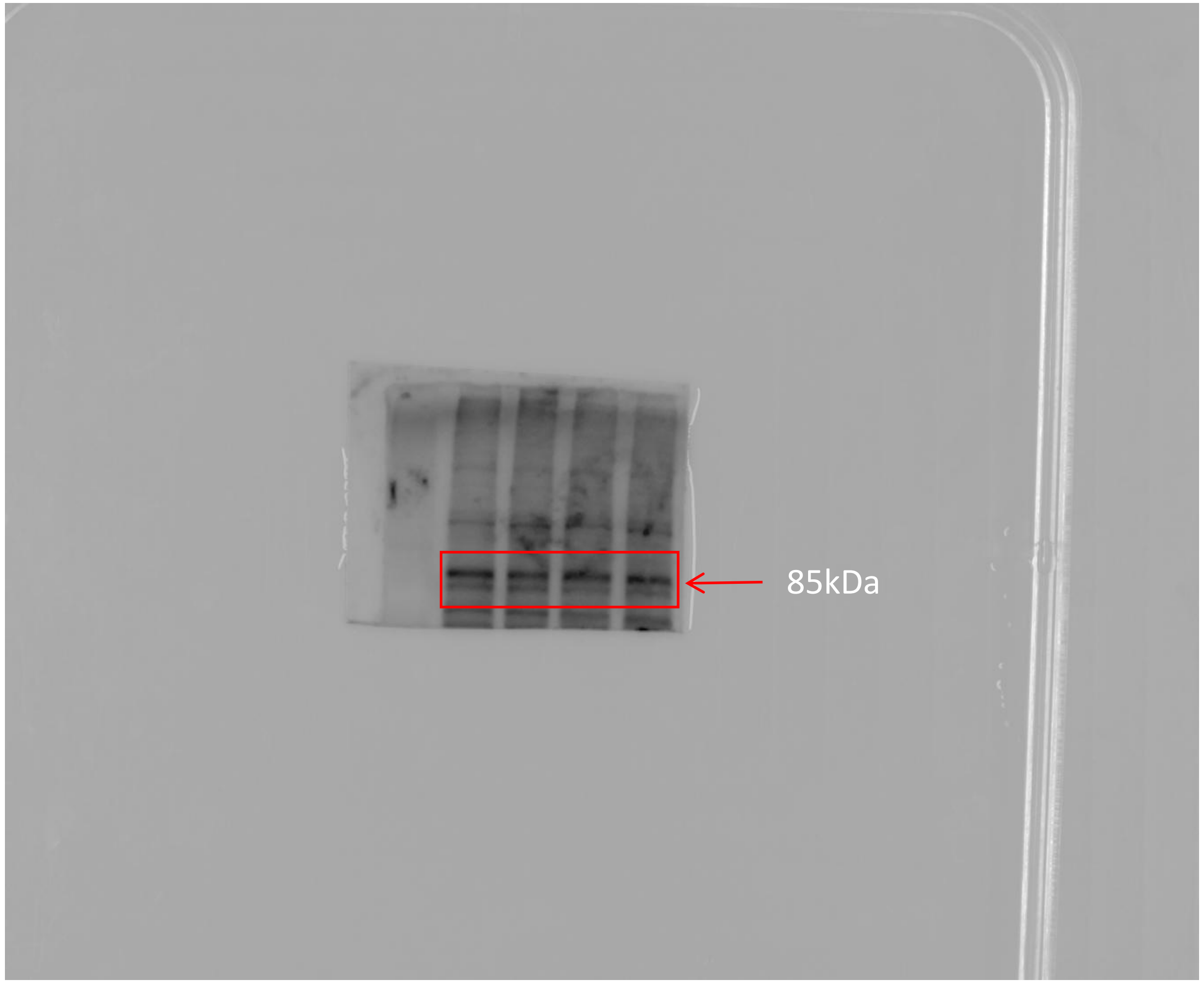

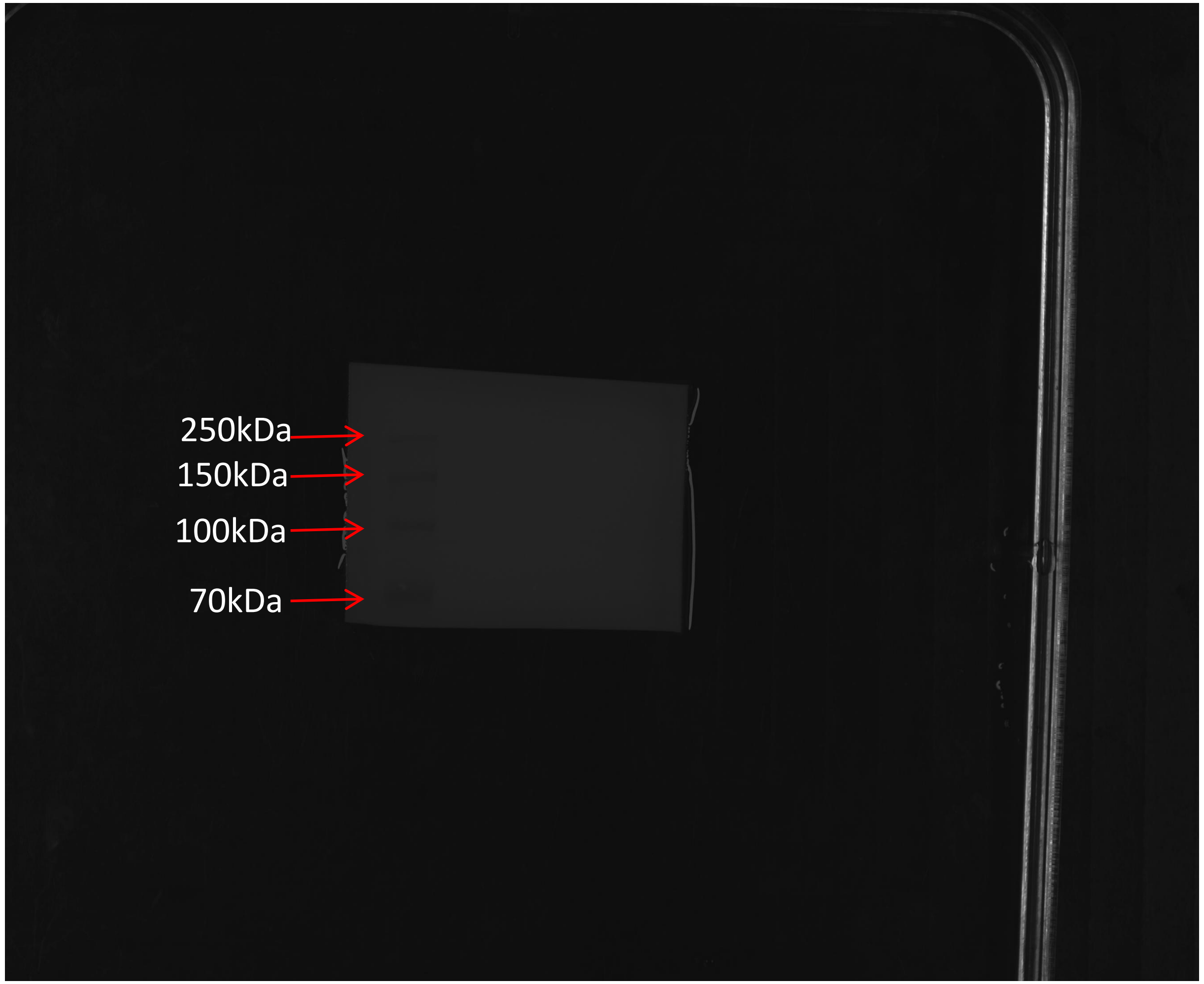

TFR

Figure S5 (3).(E) Western blot analysis of GPX4、FTH1、 NCOA4 and Drp1 proteins in mock-infected, virus-infected cells with or without the transfection of siTFR or siNC at 48 h.

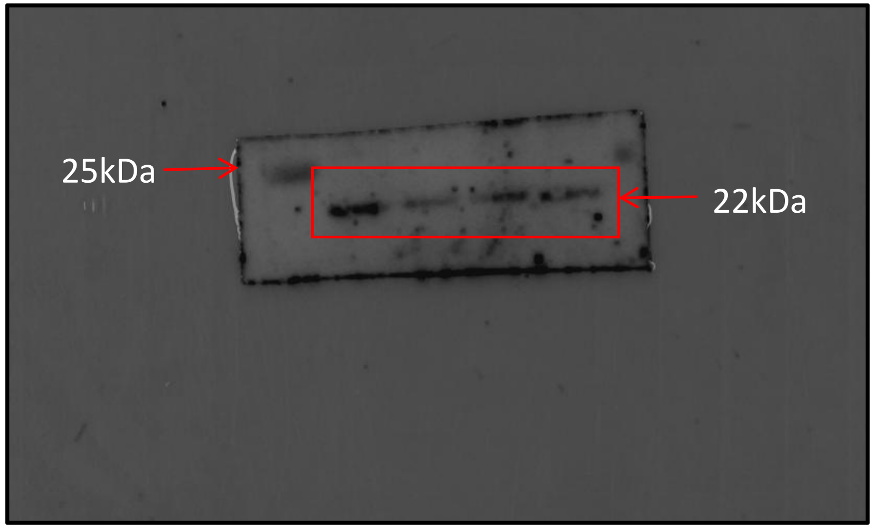

FTH1

Figure S5 (3).(E) Western blot analysis of GPX4、FTH1、NCOA4 and Drp1 proteins in mock-infected, virus-infected cells with or without the transfection of siTFR or siNC at 48 h.

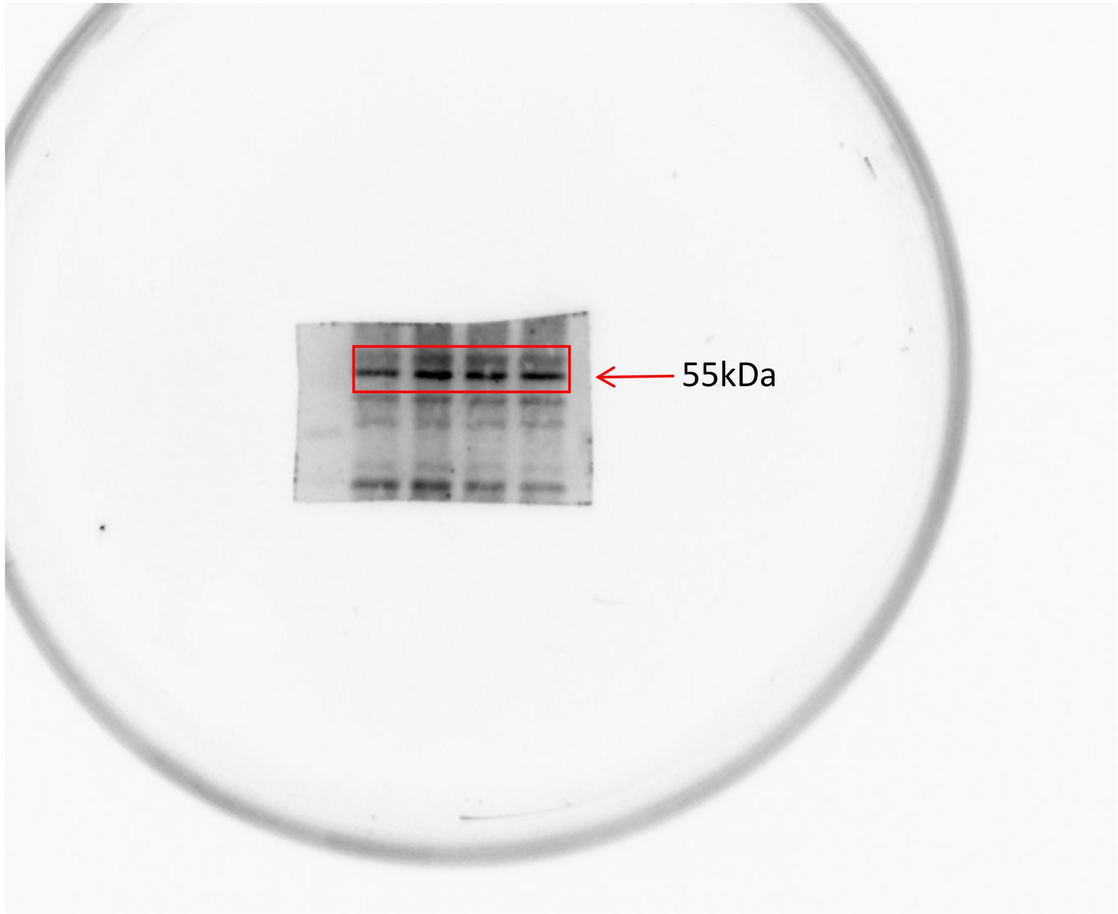

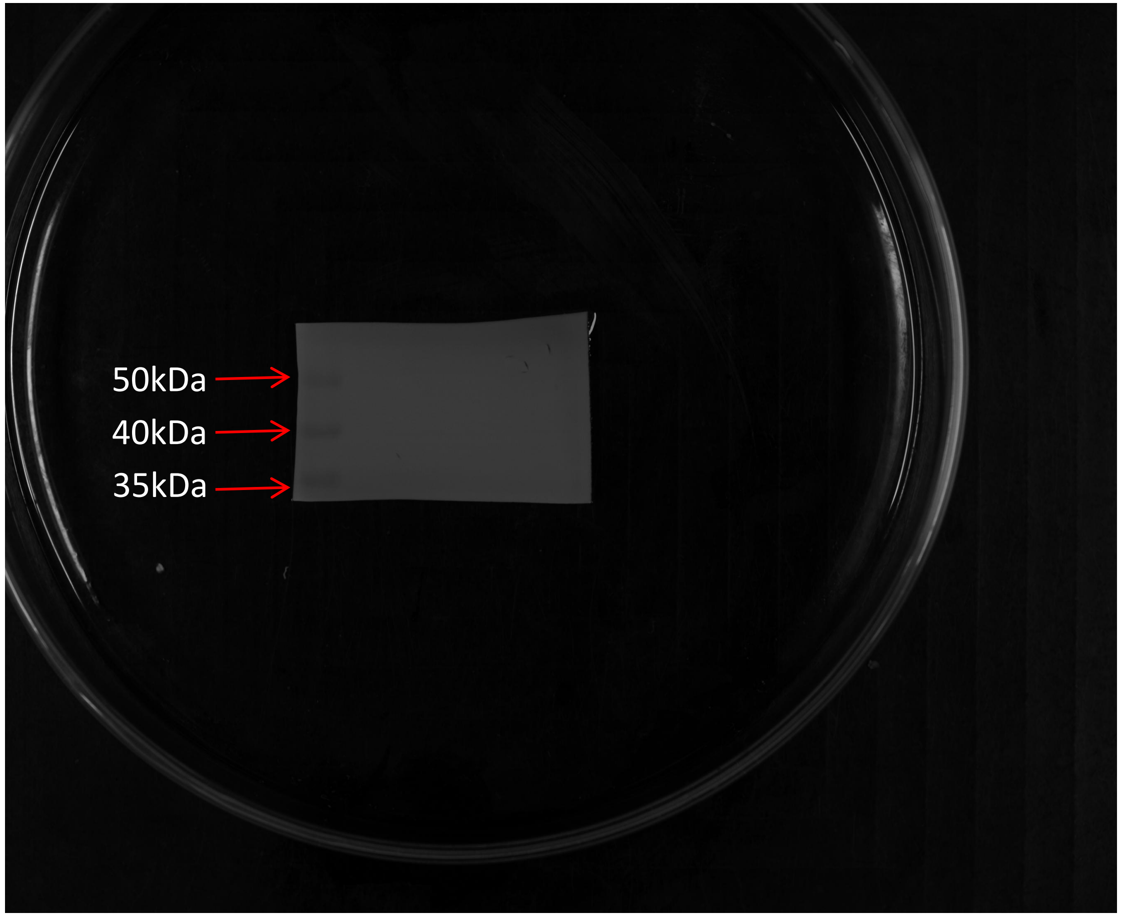

ATG5

Figure S5 (3).(E) Western blot analysis of GPX4、FTH1、NCOA4 and Drp1 proteins in mock-infected, virus-infected cells with or without the transfection of siTFR or siNC at 48 h.

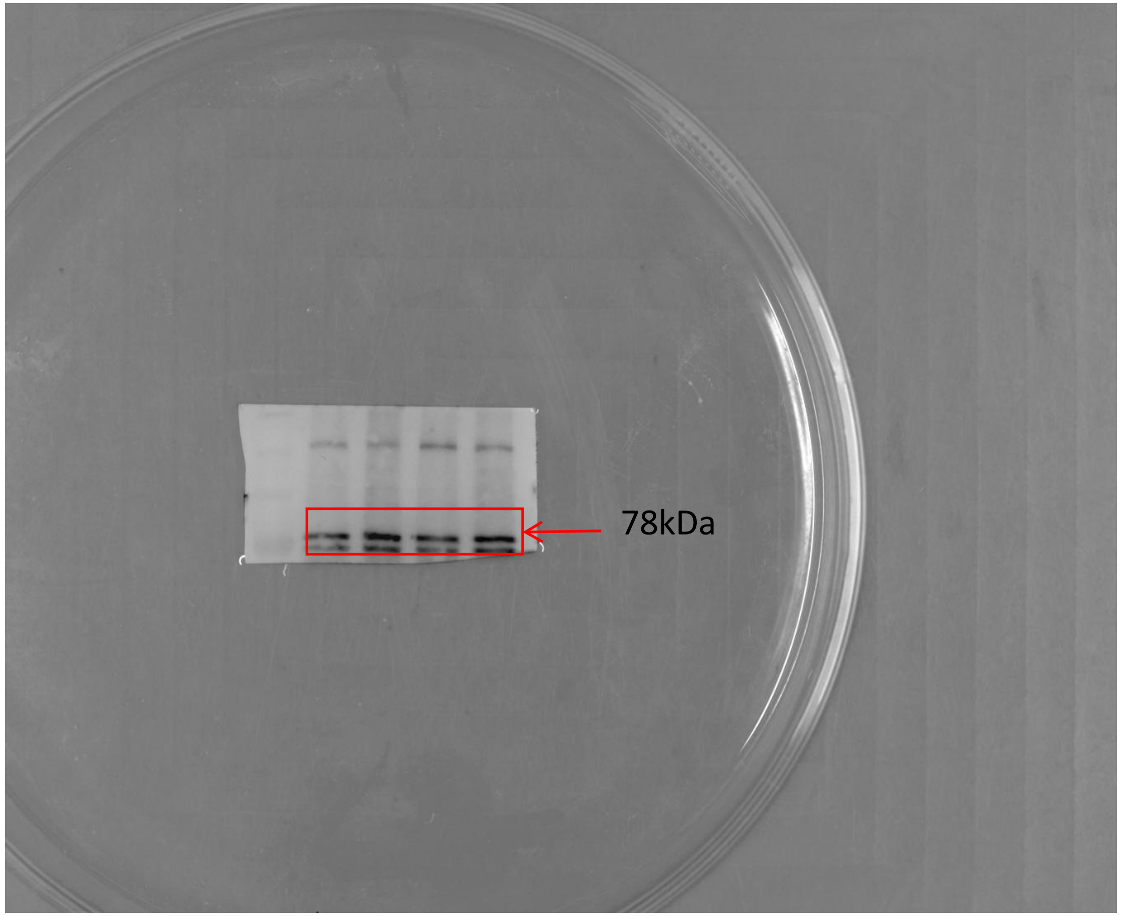

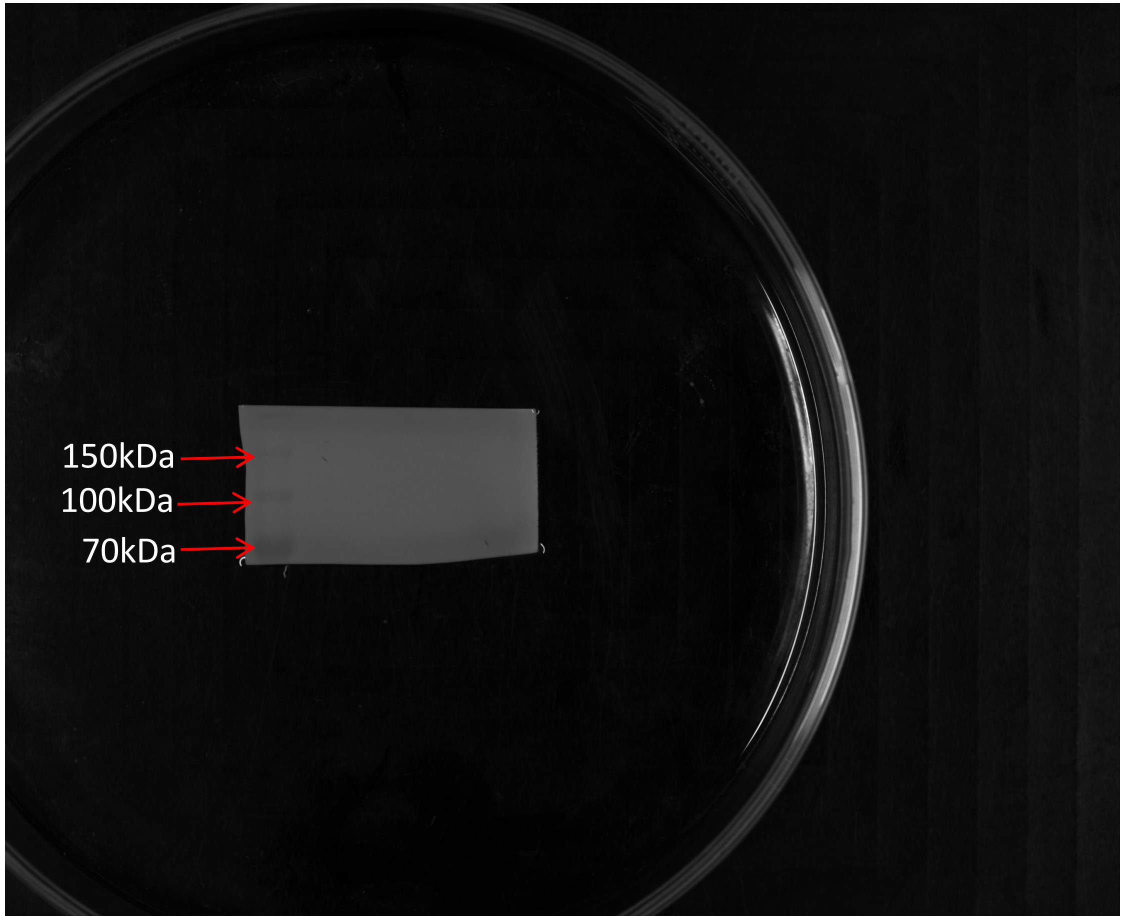

DRP1

Figure S5 (3).(E) Western blot analysis of GPX4、FTH1、NCOA4 and Drp1 proteins in mock-infected, virus-infected cells with or without the transfection of siTFR or siNC at 48 h.

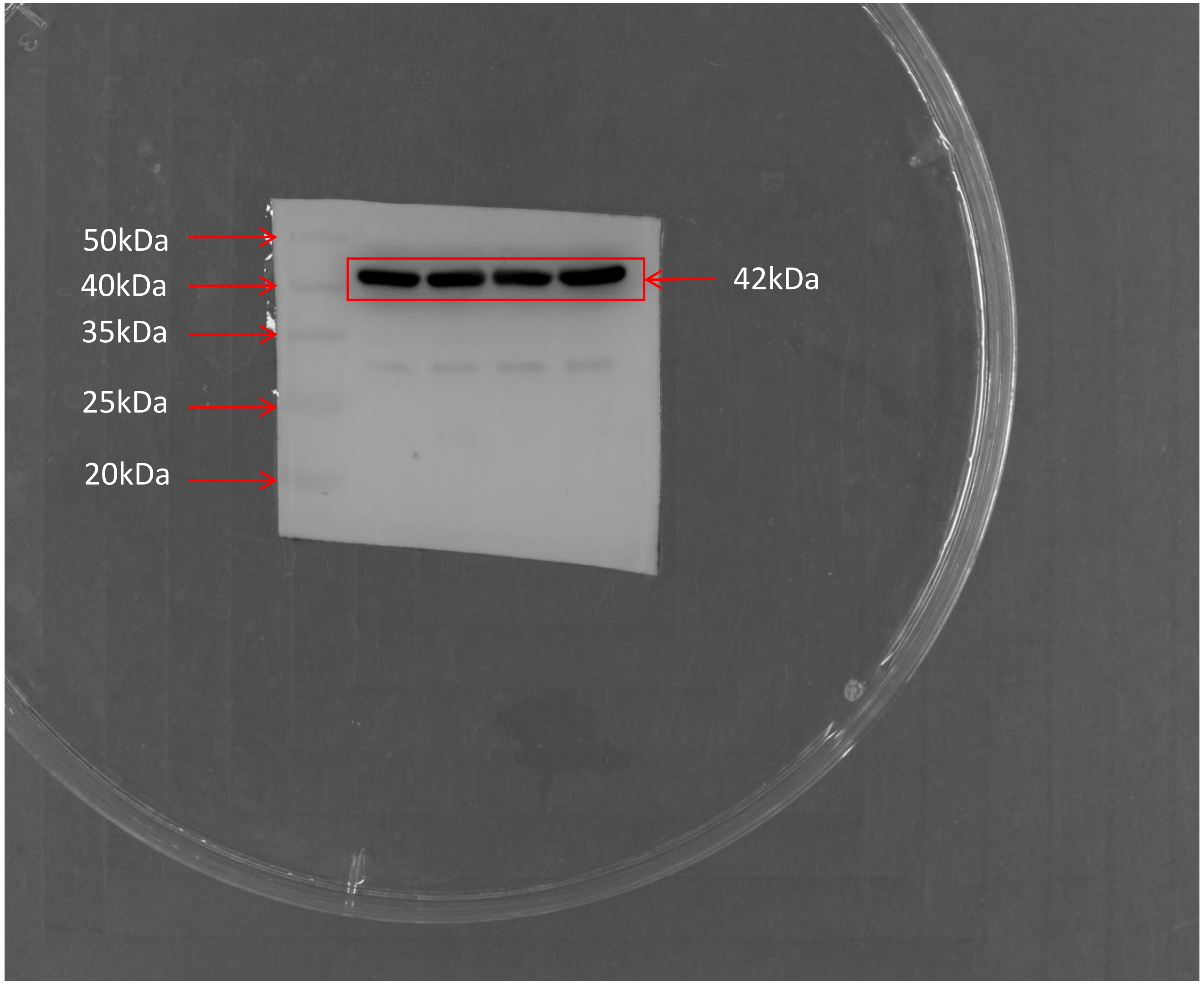

β-ACTIN
