# Supplementary material for: Deferoxamine Inhibits Canine Parvovirus by Suppressing Ferroptosis and Viral Replication
Source: Vet Sci. 2025 Dec 12;12(12):1192. doi: 10.3390/vetsci12121192 (PMC12737514; doi:10.3390/vetsci12121192)
Supplement: Supplementary file 1 [file vetsci-12-01192-s001.zip › Supplementary Methods S1 and S2.pdf]

Supplementary Methods S1. Calculation of MDA content

Intracellular MDA was quantified using a TBA-based kit. Absorbance was measured at 535 nm and converted to concentration using the MDA standard curve:

$$A_{535} = k \cdot C_{\mathrm{MDA}} + b$$

Where A_{535} is absorbance at 535 nm, MDA C_{MDA} is MDA concentration (nmol/mL), and k and b are the slope and intercept of the standard curve. Thus:

$$C_{\mathrm{MDA}} = \frac{A_{535} - b}{k}$$

MDA levels were normalized to protein content and expressed as nmol/mg protein:

$$\mathrm{MDA\ (nmol/mg\ protein)} = \frac{C_{\mathrm{MDA}} \times V_{\mathrm{rxn}}}{P_{\mathrm{sample}}}$$

where V_{rxn} is the reaction volume corresponding to the sample (mL), and sample P_{sample} is total protein amount (mg) determined by BCA assay. Reagent blank values (no sample) were subtracted before calculation.

Supplementary Methods S2. Calculation of GSH/GSSG ratio

Total glutathione (T-GSH) and GSSG were measured at 412 nm and calculated from their standard curves:

$$A_{412} = k \cdot C + b \quad \Rightarrow \quad C = \frac{A_{412} - b}{k}$$

Reduced glutathione (GSH) was derived as:

$$C_{\mathrm{GSH}} = C_{\mathrm{T\text{-}GSH}} - 2C_{\mathrm{GSSG}}$$

The redox ratio was calculated as:

$$\mathrm{GSH/GSSG\ ratio} = \frac{C_{\mathrm{GSH}}}{C_{\mathrm{GSSG}}}$$

All glutathione values were normalized to protein content:

$$\mathrm{GSH\ (nmol/mg\ protein)} = \frac{C_{\mathrm{GSH}} \times V_{\mathrm{rxn}}}{P_{\mathrm{sample}}}$$

$$\mathrm{GSSG\ (nmol/mg\ protein)} = \frac{C_{\mathrm{GSSG}} \times V_{\mathrm{rxn}}}{P_{\mathrm{sample}}}$$

Blank reactions and standards were included in each assay, and blank values were

subtracted prior to calculation.
