# Supplementary material for: Deferoxamine Inhibits Canine Parvovirus by Suppressing Ferroptosis and Viral Replication
Source: Vet Sci. 2025 Dec 12;12(12):1192. doi: 10.3390/vetsci12121192 (PMC12737514; doi:10.3390/vetsci12121192)
Supplement: Supplementary file 1 [file vetsci-12-01192-s001.zip › Table S1. The primers used in this study.pdf]

Table S1. The primers used in this study

**Primers**	**Primer Sequences (5′-3′)**	**Product Size (bp)**	**Gene accession No.**	**Reference**
GPX4	GPX4-F：CCCCTGGGTCCGTGTATT<br>GPX4-R：GAACCTACCAGGGGCTCTTC	195	XM_045042654.1	This study
ACSL4	ACSL4-F:<br>AGCCTTGGGACCAGAGAGAT<br>ACSL4-R:<br>TCCCAATGCAGTGAGTCCAC	156	XM_045050317.1	This study
TFR	TFR-F：TTCAGGTCAAAGGCAGTGCT<br>TFR-R：GCATGGACCAGTCTACCAGT	136	NC_001806	This study
FTH	FTH-F：AAAGCCACATCATCACGGTC<br>FTH1-R：CTCCTTCAGCACAGATCCCT	140	NC_058377.1	This study
ATG5	ATG5-F:<br>GACCTTCTGCACTGTCCATCT<br>ATG5-R: AATCCCATCCAGAGCTGCTT	140	XM_003986419.6	This study
NCOA4	NCOA4-F：<br>TCAGAGCTTACTGCCCTAGGAT<br>NCOA4-C-R：<br>AGAAAGTGTGGGGGAACCTG	186	XM_045040323.1	This study
Drp1	Drp1-F: CCGACATCATCCAGTTGCCT<br>Drp1-F: AGAGGTCTCCGGGTGACAAT	130	XM_023256915.2	This study
β-ACTIN	β-ACTIN- F:<br>ATATTGCTGCGCTCGTGGTC<br>β-ACTIN-R：<br>GAGTCCTTCTGGCCCATACC	145	XM_006941899.4	This study
