# Supplementary figures and images for: Deferoxamine Inhibits Canine Parvovirus by Suppressing Ferroptosis and Viral Replication
Source: Vet Sci. 2025 Dec 12;12(12):1192. doi: 10.3390/vetsci12121192 (PMC12737514; doi:10.3390/vetsci12121192)

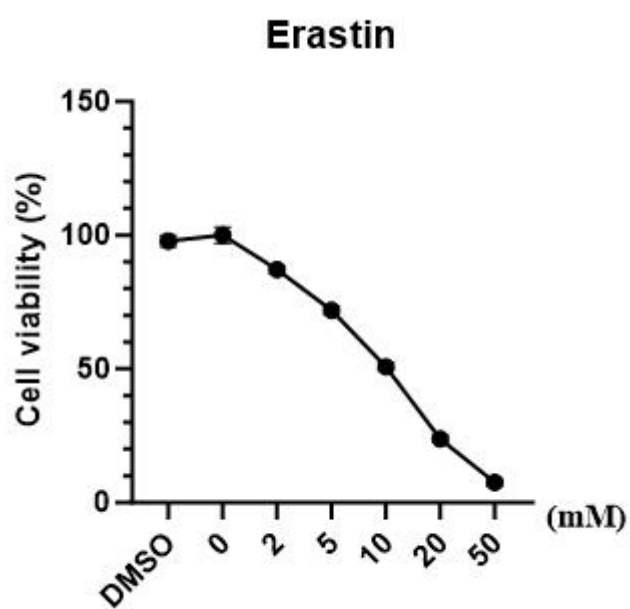

**Figure S4.** Dose–response analysis of erastin cytotoxicity in CRFK cells by CCK-8 assay.

Supplement: Supplementary file 1 [file vetsci-12-01192-s001.zip › Figure S4.pdf]
